# Comparative Efficacy and Acceptability of Pharmaceutical Management for Adults With Post-Traumatic Stress Disorder: A Systematic Review and Meta-Analysis

**DOI:** 10.3389/fphar.2020.00559

**Published:** 2020-05-08

**Authors:** Zhen-Dong Huang, Yi-Fan Zhao, Shuang Li, Hui-Yun Gu, Lu-Lu Lin, Zhi-Yan Yang, Yu-Ming Niu, Chao Zhang, Jie Luo

**Affiliations:** Center for Evidence-Based Medicine and Clinical Research, Taihe Hospital, Hubei University of Medicine, Shiyan, China

**Keywords:** post-traumatic stress disorder, pharmacotherapy, selective serotonin reuptake inhibitors, efficacy, core symptoms, all-cause discontinuation

## Abstract

The current clinical guidelines on post-traumatic stress disorder (PTSD) recommend selective serotonin reuptake inhibitors (SSRIs) and serotonin and norepinephrine reuptake inhibitors (SNRIs) of drugs. However, there is uncertainty about the efficacy of other drugs and selecting which treatments work best for which patients. This meta-analysis evaluated efficacy and acceptability of pharmaceutical management for adults with PTSD. Randomized-controlled trials, which reported active comparators and placebo-controlled trials of pharmaceutical management for adults with PTSD, from the Ovid Medline, EMBase, CENTRAL, PsycINFO, Ovid Health and Psychosocial Instruments, and ISIWeb of Science, were searched until June 21, 2019. In terms of efficacy, all active drugs demonstrated superior effect than placebo (SMD = −0.33; 95% CI, −0.43 to −0.23). The medications were superior to placebo in reducing the symptom of re-experiencing, avoidance, hyperarousal, depression, and anxiety. For acceptability, medicine interventions for PTSD showed no increase in all-cause discontinuation compared with placebo. Nevertheless, in terms of safety, medicine interventions indicated a higher risk of adverse effect compared with placebo (RR = 1.47, 95% CI: 1.24 to 1.75). Compared with placebo, the SSRIs and atypical antipsychotics drugs had significant efficacy whether in patients with severe or extremely severe PTSD status. However, only atypical antipsychotics (SMD = −0.29, 95% CI: −0.48 to −0.10) showed superior efficacy than placebo in veterans. Medication management could be effective in intervention of PTSD, which demonstrated a sufficient improvement in the core symptoms. This meta-analysis supports the status of SSRIs and SNRIs as recommended pharmacotherapy. However, patients with different clinical characteristics of PTSD should consider individualized drug management.

## Introduction

Post-traumatic stress disorder (PTSD) is a mental disorder that can occur after a person has experienced a traumatic event, such as physical abuse, sexual relationship violence, combat exposure, witnessing death or serious injury (Association, 2013). In Diagnostic and Statistical Manual of Mental Disorders (DSM)-5, PTSD is characterized by intrusion, avoidance, hyperarousal, and negative thinking in cognition and mental; all these characteristics have a certain degree of impact on a patient’s life, occupation, and interpersonal dysfunction (Association, 2013). Patients with lifetime PTSD have developed one comorbid psychiatric disorder (Maher et al., 2006; Association, 2013; Rytwinski et al., 2013), such as depression, dissociation, anxiety, and sleep disturbance. As of 2017, a survey (Koenen et al., 2017) estimated the cross-national lifetime prevalence of PTSD at 3.9% and about 5.6% of the population were exposed to trauma events. In comparison, the National Comorbidity Survey Replication (NCS-R) (Kessler et al., 2005) estimated the lifetime prevalence of PTSD among American adults in 2005 (Office, 2012) at 6.8%. The costs of managing PTSD are substantial. In 2012 (Committee on the Assessment of Ongoing Efforts in the Treatment of Posttraumatic Stress et al., 2014), the Department of Defense (DOD) and Veterans Affairs (VA) spent about USD 3 billion and USD 294 million for PTSD treatments of veterans and related service members, respectively. PTSD brings serious health-related and economic burden for patients and society.

A meta-analysis (Bromis et al., 2018) of structural magnetic resonance imaging (MRI) studies found that PTSD is associated with a decreased volume of hippocampus and structural brain abnormalities. Other reports (Geracioti et al., 2001; Milani et al., 2017) have demonstrated that PTSD patients have a greater central nervous system (CNS) noradrenergic activity under baseline conditions. PTSD is characterized by a series of neuroendocrine symptoms that may be responsive and sensitive to medication. The efficacy of selective serotonin reuptake inhibitor (SSRIs) was affirmed in previous meta-analyses (Stein et al., 2006; Hoskins et al., 2015; Puetz et al., 2015; Gu et al., 2016; Lee et al., 2016). At present, the American Psychological Association (APA) guideline (Association, 2017) suggests the use of fluoxetine, paroxetine, sertraline, and venlafaxine. Although pharmacological treatments (Association, 2017) are currently considered as an important part of clinical guidelines of PTSD management, only sertraline and paroxetine drugs are approved for PTSD by the Food and Drug Administration (FDA) to date. There is insufficient evidence to recommend for or against offering risperidone and topiramate. Nevertheless, there is no recommended first-line treatment drug for PTSD because of sufficient evidence from comparative effectiveness studies in the APA guideline (Association, 2017). More importantly, the APA guideline also indicated future research must assess the effectiveness of treatment for specific groups, such as gender differences, racial or cultural groups, and persons exposed to a particular type and severity of trauma (e.g., combat trauma, sexual assault, and community violence).

Although there are a few studies that have provided the most effective interventions for particular patients under specific conditions, they do not address the awkward situation of choosing an appropriate drug for different types of PTSD patients (Association, 2017) in clinical practice. Considering the uncertainty of existing evidence and the lack of information on a particular type of trauma (e.g., the severity of trauma), clinical guidelines have not yet provided a clear intervention scheme for PTSD management. This meta-analysis evaluated efficacy, acceptability, and safety of pharmacological treatments while considering patients’ clinical characteristics; it provides the latest evidence that can help make decisions for pharmaceutical management of PTSD in adults.

## Methods

### Search Strategy and Selection Criteria

We used the guidelines from the Preferred Reporting Items for Systematic Review and Meta-Analyses (PRISMA) statement (Moher et al., 2009). All studies were obtained by searching the Ovid Medline, EMBase, CENTRAL, PsycINFO, Ovid Health and Psychosocial Instruments, and ISIWeb of Science for articles that were published until June 21, 2019.

Four reviewers (Z-DH, H-YG, Y-FZ, and SL) independently assessed the abstracts and potentially eligible articles identified during literature selection. Discrepancies were resolved in discussions. If necessary, a final reviewer (CZ) was involved when faced with a disagreement. Detailed search strategies are shown in **Supplemental Method 1**.

### Inclusion and Exclusion Criteria

Studies were included when they met the following criteria: (1) Adults (≥18 years old) with a primary diagnosis of PTSD according to diagnostic criteria (DSM-III, DSM-III-R, DSM-IV, DSM-IV-TR, DSM-V, or ICD-10 (santé Omdl et al., 1992) (2) Interventions: pharmacological treatments whether oral or intravenous infusion for adults with PTSD, such as monotherapy, adjunctive, or augmentation interventions; (3) Comparisons: placebo or other active drugs; (4) Outcomes: efficacy (change in PTSD total symptoms using clinician rating scales or interview instruments (Association, 2018); reduction rate of core symptoms, including re-experiencing, avoidance, and hyperarousal; reduction rate of other symptoms, including depression and anxiety) and acceptability (all-cause discontinuation and discontinuation due to adverse effects). All-cause discontinuation was used as a measure for the acceptability of treatments because it encompasses efficacy and tolerability; (5) type of studies: randomized controlled trials (RCTs).

Studies were excluded for the following reasons: (1) The presence of a schizophrenia, schizoaffective disorder or bipolar disorder in particular cases participants with current depression or anxiety were included provided that their sub-symptom was secondary to PTSD; (2) Cognitive disorder or at immediate risk of suicide; (3) Prevention or prevention of relapse trials; (4) Combination therapy with two or more drugs as main intervention, or psychological treatment combined with medication; (5) Data were not available or not convertible from the original research; (6) Duplicate publication.

### Data Extraction and Quality Assessment

Information and data were extracted by four independent authors (ZDH, HYG, FYZ, and ZYY); a final investigator (CZ) proofread and handled any arguments. To reduce the heterogeneity of research in a variety of clinical measurement tools (Fervaha et al., 2015), we used clinician rating scales or interview instruments (Association, 2018) to assess PTSD total symptoms. We only used the clinically administered PTSD scale (Association, 2013) to assess the severity of trauma (Weathers et al., 2001), which is defined as few symptoms (0–19), mild PTSD (20–39), moderate PTSD (40–59), severe PTSD symptomatology (60–79), and extreme PTSD symptomatology (≥80). Objective drugs that we study were added to an ongoing pharmacotherapy regimen that is, adjunctive treatment. To avoid over-optimistic estimates of the efficacy, data were extracted from the intention to treat (ITT) sample whenever possible (Gupta, 2011). We used the change values from the baseline as much as possible in all the continuous outcomes. If change values from the baseline were not mentioned, we used a comparison of final measurements according to Cochrane Handbook (Higgins and Green, 2011), which is a randomized trial estimating the same baseline value in theory. We prioritized the results of 8–12 weeks after drug treatment if a study reported different stages of treatment outcomes to reduce the associated heterogeneity impact. Two authors (ZDH and HYG) independently assessed the risk of bias in accordance with the Cochrane risk of bias tool (Higgins and Green, 2011).

### Statistical Analysis

We performed pooled risk ratios (RR) with 95% confidence intervals (CI) using the Mantel-Haenszel statistical method for dichotomous data. Continuous data (Hedges, 1981) were analysed as the mean difference (MD) or standardized mean difference (SMD) with 95% CI. The SMD was used when the studies assessed the same outcome but different unit measurements; otherwise, MD was employed. SD was obtained from standard errors (SE) and CI for group means by appropriate statistical methods based on Cochrane Handbook (Higgins and Green, 2011). If studies only reported the median and range of the samples or the first and third quartiles, we estimated the sample mean and SD (Wan et al., 2014; Luo et al., 2018).

The I^2^ statistics (Higgins and Green, 2011) were used to assess the heterogeneity of each analysis (Higgins and Green, 2011). When I^2^ < 40%, a fixed-effect model was used. If I^2^ ≥40, we assumed that there was statistical heterogeneity, therefore the pooled effect size was calculated by the random effects model. We performed pooling analysis of different outcomes based on placebo-controlled and active-comparators trials. Subgroup analyses were based on different classifications of pharmacological mechanisms and specific drug branches and therapeutic regimens (monotherapy and adjunctive drugs). We also conducted stratified analyses to explore the special efficacy of different drugs for patient ages (older adults, older than 60 years and non-older adults), gender (male and female), races, populations (veterans or civilians), and severity of trauma based only on CAPS scores (severe and extreme PTSD symptomatology); the outcome of changes in PTSD total symptoms, which were based on a comparison of active drugs with placebo, was used. Meta-regression analysis was performed to explore the effects of modifiers on the efficacy of overall PTSD symptoms. The analysis included mean age at onset, baseline severity, publication year, and sponsorship. The trim-and-fill method base on funnel plots (Duval and Tweedie, 2000) was used to evaluate the possible publication bias of the efficacy of the drug. Funnel plots were used only for at least 10 studies to ensure adequate test performance (Higgins and Green, 2011). All statistical analyses were performed using R 3.5.2 software.

## Results

### Literature Identification

**Figure 1:**
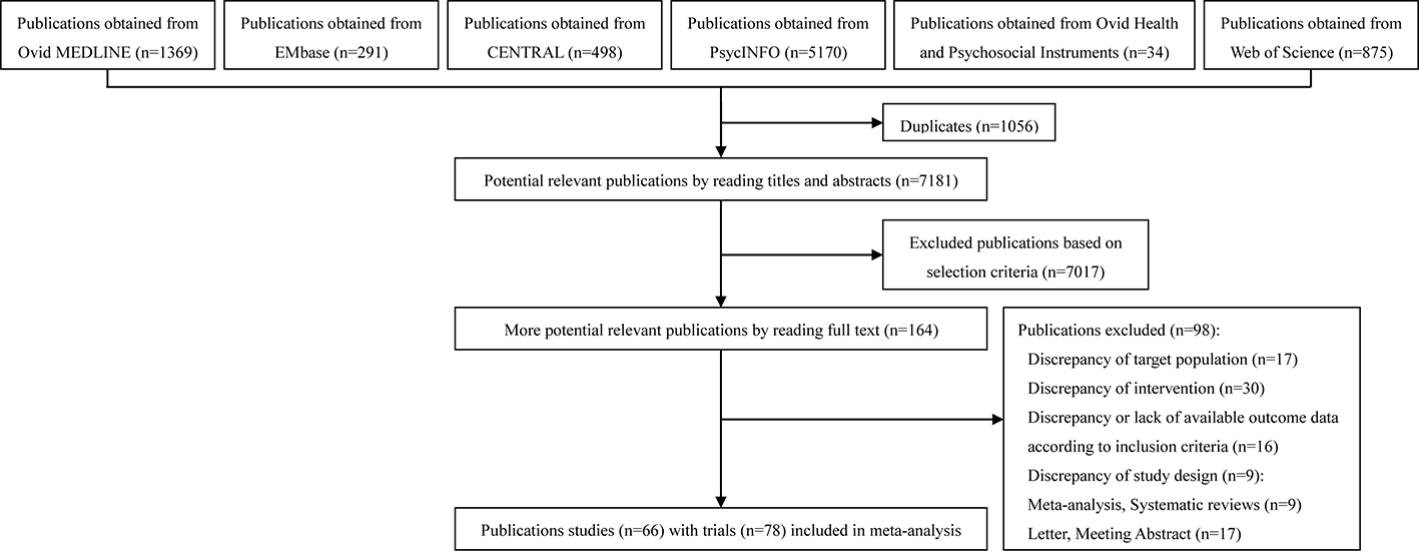
Study selection.

The Ovid Medline, Ovid EMBASE, CENTRAL, and Web of Science were systematically searched until June 21, 2019. The search resulted in 8,237 articles. After initial evaluation, 1,056 studies were removed for being duplicates, 7,017 for being irrelevant (as determined by reading the title and abstracts), and 66 studies for reasons determined by reading the full text. The final 66 studies, with 78 trials, were used in this study. **Figure 1** shows the work flow for the selection of studies.

### Study Characteristics

**Table 1** summarizes patient characteristics (i.e., author, sample, age, gender, race, population, severity of trauma, type of trauma, baseline score, diagnostic criteria, and drug dosage during treatment). In total, there were 14 trials of all-male populations, Three RCTs of drug treatment for all-female patients, and the rest were of mixed gender population. Although the DSM-5 indicates four core symptoms for PTSD, all of included studies only evaluated three core symptoms of PTSD (i.e., re-experiencing, avoidance, and hyperarousal) and other symptoms (depression and anxiety), therefore, these chosen symptoms were investigated to select the best pharmaceutical management regimens in this study. The average age range of population in all trials was 20 to 60 years. A total of 31 trials involving 1,987 veterans (1,831 patients combat-related PTSD) were included in PTSD pharmacological treatments trials. The remaining trials were contained in the population for civilians or mixed (civilians and veterans) (**Table 1**). Among the 66 included studies (Davidson et al., 1990; Kosten et al., 1991; Katz et al., 1994; van der Kolk et al., 1994; Baker et al., 1995; Connor et al., 1999; Hertzberg et al., 1999; Brady et al., 2000; Hertzberg et al., 2000; Butterfield et al., 2001; Davidson et al., 2001; Marshall et al., 2001; Tucker et al., 2001; Martenyi et al., 2002; Stein et al., 2002; Zohar et al., 2002; Davidson et al., 2003; Hamner et al., 2003; Monnelly et al., 2003; Tucker et al., 2003; Davis et al., 2004; McRae et al., 2004; Reich et al., 2004; Akuchekian et al., 2004; Bartzokis et al., 2005; Davidson et al., 2006a; Davidson et al., 2006b; Neylan et al., 2006; Padala et al., 2006; Spivak et al., 2006; Becker et al., 2007; Davidson et al., 2007; Friedman et al., 2007; Lindley et al., 2007; Marshall et al., 2007; Martenyi et al., 2007; Raskind et al., 2007; Tucker et al., 2007; van der Kolk et al., 2007; Davis et al., 2008a; Davis et al., 2008b; Rothbaum et al., 2008; Hamner et al., 2009; Krystal et al., 2011; Mathew et al., 2011; Panahi et al., 2011; Yeh et al., 2011; Carey et al., 2012; Raskind et al., 2013; Ahmadpanah et al., 2014; Baniasadi et al., 2014; Batki et al., 2014; Naylor et al., 2015; Back et al., 2016; Mahabir et al., 2016; Petrakis et al., 2016; Ramaswamy et al., 2016; Villarreal et al., 2016; Dunlop et al., 2017; Li et al., 2017; Ramaswamy et al., 2017; Rezaei Ardani et al., 2017; Suris et al., 2017; Brunet et al., 2018; Hodgins et al., 2018; Raskind et al., 2018), six (Kosten et al., 1991; Marshall et al., 2001; Tucker et al., 2003; Davidson et al., 2006b; Martenyi et al., 2007; Ahmadpanah et al., 2014) conducted three separate trial arms (a comparison of two active drugs with placebo). Our research included 78 published double-blind, parallel RCTs. This meta-analysis involved 70 RCTs, which involved a comparison of 31 active drugs and placebo, and also extended to eight active-comparators trials (Kosten et al., 1991; Marshall et al., 2001; Tucker et al., 2003; McRae et al., 2004; Davidson et al., 2006b; Spivak et al., 2006; Martenyi et al., 2007; Ahmadpanah et al., 2014), comparing 12 active drugs, to explore the discovery of differences among different drugs. According to the pharmacological effects of drug classification of pharmacological mechanisms and abbreviation, as shown in **Supplemental Table 1**, 8,083 patients diagnosed with PTSD were randomly assigned to active drug or placebo group and had at least one post baseline evaluation in the analysis. **Supplemental Table 2** shows 44 of 66 studies or 66% obtained funding support from pharmaceutical companies.

**Table 1:** Summary of included clinical trials and patient characteristics.

Study	Sample (I/C)	Mean Age (SD)	Gender (Male)	Race (White)	Population	Type of trauma	Diagnostic criteria	Baseline score	Drug dose (mg/d)	During of treatment (week)
Ahmadpanah et al., 2014	33/34	36.15 (6.53)	50 (74.63%)	NR	Mixed	Mixed	DSM-IV TR	(M.I.N.I.) 7 (0.35)	Prazosin 1–15 mg/d versus hydroxyzine 10–100	8
Ahmadpanah et al., 2014	33/33	35.2 (6.61)	47 (71.21%)	NR	Mixed	Mixed	DSM-IV TR	(M.I.N.I.) 7 (0.28)	Prazosin 1–15 mg/d versus placebo	8
Ahmadpanah et al., 2014	34/33	35.18 (6.08)	47 (70.15%)	NR	Mixed	Mixed	DSM-IV TR	(M.I.N.I.) 7 (0.45)	Hydroxyzine 10–100 mg/d versus placebo	8
Akuchekian et al., 2004	34/33	39.8 (4.19)	67 (100%)	NR	Veterans	All combat-related	DSM-IV	CAPS: 49.81 (8.42)	Topiramate (50–500 mg/d) versus placebo	12
Back et al., 2016	13/14	49.0 (8.2)	26 (96.3%)	8	Veterans	Military(combat5, non-combat 9),civilian-related events 13	DSM-IV	CAPS: 63.88 (22.65)	Fixed dose n-acetylcysteine 2400 versus placebo	8
Baker et al., 1995	56/58	43.98 (7.19)	92 (80.75%)	NR	Mixed	Mixed	DSM-III-R	CAPS: 83.4 (17.95)	Brofaromine, titrated up to 150 mg versus placebo	12
Baniasadi et al., 2014	18/19	48.16 (3.55)	37 (100%)	NR	Veterans	All combat-related	DSM-IV-TR	PCL-M 55.94 (7.65)	Pregabalin 75–300 versus placebo	6
Bartzokis et al., 2005	33/32	51.6 (4.2)	65 (100%)	44	veterans	All combat-related	DSM-IV	CAPS: 100.43 (13.96)	Risperidone 1 to 3 mg versus placebo	16
Batki et al., 2014	14/16	49.98 (13.1)	28 (93.3%)	16	Veterans	22 Combat-related	DSM-IV-TR	CAPS: 78.29 (16.55)	Topiramate (25–300) versus placebo	12
Becker et al., 2007	18/10	50.39 (7.46)	22 (79%)	8	Mixed	Mixed	DSM-IV	NR	Bupropion sr 100–300 mg/d versus placebo	8
Brady et al., 2000	94/93	39.85 (10.1	50 (26.74%)	158	Mixed	Mixed	DSM-III-R	CAPS: 75.86 (17.56)	Sertraline 133.3 (25–200) versus placebo	12
Brunet et al., 2018	30/30	39.4 (11.38)	25 (41.7%)	42	Mixed	Mixed	DSM-IV-TR	CAPS: 73.56 (15.94)	0.67 mg/kg of conventional (short-acting) propranolol, plus 1.0 mg/kg of long-acting propranolol versus placebo	6
Butterfield et al., 2001	10/5	43.2 (14.73)	1 (6.67%)	8	Mixed	Mixed	DSM-IV	SI-PTSD 41.77 (9.43)	Olanzapine 5–20 mg/d versus placebo	10
Carey et al., 2012	14/14	40.75 (11.59)	11 (39%)	NR	Civilians	Civilians	DSM-IV	CAPS: 80.5 (13.64)	olanzapine 5–15 mg/d versus placebo	8
Connor et al., 1999	27/27	37.17 (2.64)	5 (9%)	50	Civilians	Mixed	DSM-III-R	DTS 76.55 (21.62)	Fluoxetine 10–60 mg/d or placebo	12
Davidson et al., 1990	25/21	49.22 (11.94)	NR	NR	Veterans	All combat-related	DSM-III	IES 34.54 (7.59)	Amitriptyline 160.7 (50 to 300) or placebo	8
Davidson et al., 2001	100/108	37.1 (10.6)	46 (22.12%)	174	Mixed	Mixed	DSM-III-R	CAPS: 73.69 (16.11)	Sertraline 146.3 (25–200) versus placebo	12
Davidson et al., 2003	17/9	46.5 (13)	11 (42.3%)	NR	Mixed	Mixed	DSM-IV	SPRINT 22.84 (5.59)	Mirtazapine (15–45) versus placebo	8
Davidson et al., 2006a	179/173	NR	NR	NR	Mixed	Mixed	DSM-IV	CAPS: 83.02 (15.24)	Venlafaxine er (37.5–300 mg/d) versus sertraline (25–200 mg/d)	12
Davidson et al., 2006a	179/179	NR	NR	NR	Mixed	Mixed	DSM-IV	CAPS: 82.8 (14.84)	Venlafaxine er (37.5–300 mg/d) versus placebo	12
Davidson et al., 2006a	173/179	NR	NR	NR	Mixed	Mixed	DSM-IV	CAPS: 81.8 (15.05)	Sertraline (25–200 mg/d) versus placebo	12
Davidson et al., 2006b	161/168	41.33 (12.58)	151 (45.9%)	NR	Mixed	Mixed	DSM-IV	CAPS: 81.97 (15.08)	Venlafaxine er (37.5–300 mg/d) versus placebo	24
Davidson et al., 2007	105/97	42.6 (11.8)	79 (34%)	NR	Mixed	Mixed	DSM-IV	CAPS: 82.55 (15.08)	Tiagabine (4–16) versus placebo	12
Davis et al., 2004	26/15	53.8 (8.1)	40 (97.6%)	22	Veterans(40) and Civilian(1)	Combat-related, 40 (97.5%)	DSM-IV	CAPS: 81.8 (18.77)	Sertraline (100–600) versus placebo	12
Davis et al., 2008a	44/41	55.2 (6.8)	2 (98%)	NR	Veterans	78 Combat-related	DSM-IV	CAPS: 76.21 (17.3)	Divalproex 2309 (1,000–3,000) versus placebo	8
Davis et al., 2008b	18/17	53.46 (7.46)	32 (91.43%)	25	Veterans	All combat-related	DSM-IV	CAPS: 85.14 (17.38)	Guanfacine (1–2 mg/d) versus placebo	8
Dunlop et al., 2017	63/65	40.5 (12.1)	0 (0%)	72	Mixed	Mixed	DSM-IV	CAPS: 76.17 (16)	Fixed dose 350 mg/d of gsk561679 versus placebo	6
Friedman et al., 2007	86/83	45.32 (10.31)	135 (80%)	120	Veterans	120 Combat-related	DSM-III-R	CAPS: 72.93 (19.41)	Sertraline (25–200 mg/day) or placebo	12
Hamner et al., 2003	19/18	52.21 (6.44)	NR	17	Veterans	All combat-related	DSM-IV	CAPS: 89.72 (18.31)	Risperidone 20–40 versus placebo	5
Hamner et al., 2009	16/13	52.38 (6.89)	28 (96.55%)	26	Veterans	Combat (n = 28) and sexual assault (n = 1)	DSM-IV	CAPS: 77.1 (22.57)	Divalproex 1196 mg (500–1,500) versus placebo	10
Hertzberg et al., 1999	10/4	43.36 (8.32)	9 (64.29%)	4	Mixed	Mixed	DSM-IV	SI-PTSD 44.29 (6.3)	Lamotrigine 380 (25–500) versus placebo	12
Hertzberg et al., 2000	6/6	46 (r:44–48)	12 (100%)	5	Veterans	All combat-related	DSM-IV	DTS 108.5 (20.09)	Fluoxetine 48 (10–60) versus placebo	12
Hodgins et al., 2018	63/65	40.5 (12)	0 (0%)	72	Civilians	NR	DSM-IV	CAPS: 74.37 (16.2)	gsk561679 versus placebo	6
Katz et al., 1994	22/23	39	34 (75.56%)	NR	Mixed	Mixed	DSM-III-R	CAPS: 81.78 (18.11)	Brofaromine 50–150 versus placebo	14
Kosten et al., 1991	19/23	39 (1.98)	42 (100%)	39	Veterans	All combat-related	DSM III	IES 33.83 (16.12)	Imipramine 225 (50–300) versus phenelzine 68 mg (15–75)	8
Kosten et al., 1991	19/18	38.51 (2.04)	37 (100%)	31	Veterans	All combat-related	DSM III	IES 31.77 (14.21)	Imipramine 225 (50–300) versus placebo	8
Kosten et al., 1991	23/18	38.56 (2.04)	41 (100%)	34	Veterans	all combat-related	DSM III	IES 34.96 (15.26)	phenelzine 68 mg (15–75)versus placebo	8
Krystal et al., 2011	133/134	54.4 (10.7)	258 (96.6%)	117	Veterans	209 combat-related events	DSM-IV	CAPS: 78.2 (14.82)	Risperidone (1–4) versus placebo	24
Li et al., 2017	36/36	46 (6.01)	63 (87.5%)	0	Mixed	Mixed	DSM-IV	IES-R 64.35 (3.9)	Fixed dose sertraline 135 mg daily versus placebo	12
Lindley et al., 2007	20/20	53.4 (0.76)	40 (100%)	25	Veterans	all combat-related	NR	CAPS: 61.55 (17.93)	Topiramate (50–200) versus placebo	7
Mahabir et al., 2016	20/21	43.41 (11.65)	11 (26.83%)	NR	Mixed	Mixed	DSM-IV	CAPS: 79.4 (20.13)	Fixed dose propranolol 1 mg/kg versus placebo	1
Marshall et al., 2001	183/186	41.8 (11.6)	119 (32.25%)	NR	Mixed	Mixed	DSM-IV	CAPS: 74.85 (15.98)	Fixed dose paroxetine (20 mg/d) versus placebo	12
Marshall et al., 2001	182/186	41.8 (11.6)	117 (31.8%)	NR	Mixed	Mixed	DSM-IV	CAPS: 74.35 (15.73)	Fixed dose paroxetine (40 mg/d) versus placebo	12
Marshall et al., 2001	183/182	41.8 (11.6)	112 (30.7%)	NR	Mixed	Mixed	DSM-IV	CAPS: 74.8 (15.84)	Fixed dose paroxetine (20 mg/d) versus paroxetine (40 mg/d)	12
Marshall et al., 2007	25/27	39.8 (11.2)	17 (32.7%)	NR	Civilians	Mixed	DSM-IV	CAPS: 83.53 (25.84)	Paroxetine 10–60 mg/d versus placebo	10
Martenyi et al., 2002	226/75	37.93 (9.33)	245 (81.4%)	273	Mixed	301 combat-related	DSM-IV	CAPS: 80.7 (15.53)	Fluoxetine 48 (10–60) versus placebo	12
Martenyi et al., 2007	163/88	41.2 (11.5)	72 (28.7%)	198	Mixed	Mixed	DSM-IV	CAPS: 77.71 (16.27)	Fixed dose 20 mg/d fluoxetine versus placebo	12
Martenyi et al., 2007	160/88	40.5 (12)	70 (28.2%)	192	Mixed	Mixed	DSM-IV	CAPS: 77.22 (15.91)	Fixed dose 40 mg/d fluoxetine versus placebo	12
Martenyi et al., 2007	163/160	40.5 (11.88)	92 (28.5%)	242	Mixed	Mixed	DSM-IV	CAPS: 78.57 (15.64)	Fixed dose 20 mg/d fluoxetine versus 40 mg/d fluoxetine	12
Mathew et al., 2011	20/19	40.8 (11.96)	16 (41%)	14	Mixed	Mixed	DSM-IV	CAPS: 73 (13.7)	GR205171 (5 mg/day) with placebo	8
McRae et al., 2004	13/13	40.27 (10.73)	6 (23%)	NR	Civilians	Mixed	DSM-IV	CAPS: 68.81 (13.53)	Nafazodone 463 (100–600) versus sertraline 153 (50–200)	12
Monnelly et al., 2003	7/8	51.35 (6.3)	15 (100%)	NR	Veterans	All combat-related	DSM-IV	PCL-M 72.47	Risperidone 0.5–2 mg versus placebo	6
Naylor et al., 2015	7/7	33.82 (4.81)	9 (64.29%)	7	Veterans	All combat-related	DSM-IV	CAPS: 86.45 (15.37)	Aripiprazole 5–20 versus placebo	10
Neylan et al., 2006	29/34	NR	NR	NR	Veterans	All combat-related	DSM-IV	CAPS: 68.34 (20.57)	Guanfacine 0.5–3 versus placebo	8
Padala et al., 2006	11/9	41.3 (NR)	0 (0%)	14	Civilians	All related to sexual assault and domestic abuse	DSM-IV	CAPS: 79.89	Risperidone (1–6) versus placebo	12
Panahi et al., 2011	35/35	45.55 (5.3)	70 (100%)	NR	Veterans	Male Iranian veterans with combat-related PTSD	DSM-IV-TR	IES-R 65.25 (4.45)	Sertraline 50–200 versus placebo	10
Petrakis et al., 2016	50/46	43.97 (13.02)	89 (92.71%)	78	Veterans	All combat-related	DSM-IV	CAPS: 73.78 (17.77)	Prazosin 14.5 (2–16) versus placebo	13
Ramaswamy et al., 2016	15/15	38.9 (11.84)	4 (13.33%)	NR	Civilians	Mostly related to sexual or physical assault	DSM-IV	CAPS: 77.65 (17.66)	Ziprasidone 40–160 versus placebo	9
Ramaswamy et al., 2017	29/30	32.7 (7.1)	57 (97%)	32	Veterans	All Combat-Related	DSM-IV	CAPS: 75.45 (12.98)	Vilazodone (10–40) versus placebo	12
Raskind et al., 2007	20/20	26 (9)	2 (5%)	26	Veterans	all Combat-Related	DSM-IV	CAPS: 77 (19.87)	Prazosin 13 (2–15) versus placebo	8
Raskind et al., 2013	32/35	30.42 (6.51)	57 (85.07%)	42	soldiers (65) and veterans (2)	All Combat-Related	DSM-IV	CAPS: 81.69 (22.72)	Prazosin 1–20 mg for men or 1–10 mg for women versus placebo	15
Raskind et al., 2018	152/152	51.85 (13.78)	297 (97.7%)	203	Veterans	All Combat-Related	DSM-IV	CAPS: 81.3 (16.3)	Prazosin (1–20 mg for men or 1–12 mg for women) versus placebo	10
Reich et al., 2004	12/9	27.86 (18–34)	21 (100%)	18	Civilians	All childhood abuse related	DSM-III-R	CAPS: 69.1 (12.54)	Risperidone (0.5–8) versus placebo	8
Rezaei Ardani et al., 2017	12/12	50.22 (5.66)	24 (100%)	NR	Veterans	All combat-related	DSM-IV-TR	PCL-M 49.5 (5.85)	Rivastigmin 3–6 mg/d versus placebo	12
Rothbaum et al., 2008	9/11	34.17 (11.10)	4 (20%)	14	Civilians	Mixed	DSM-IV	CAPS: 57.76 (20.8)	Risperidone (0.5–3) versus placebo	8
Spivak et al., 2006	20/20	40.64 (9.42)	15 (53.57%)	NR	Civilians	All motor vehicle accident-related	DSM-IV	CAPS: 77.8 (16)	Fixed dose Reboxetine (8 mg/d) versus fluvoxamine (150 mg/d)	8
Stein et al., 2002	10/9	53.26 (7.44)	19 (100%)	NR	Veterans	All combat-related	DSM-IV	CAPS: 85.11 (19.03)	Olanzapine 15 (10–20) versus placebo	8
Suris et al., 2017	26/28	37.5 (14.15)	54 (100%)	36	Veterans	all combat-related	DSM-IV-TR	PCL 55.85 (10.88)	Dexamethasone 0.15 mg/kg versus placebo	2
Tucker et al., 2001	151/156	40.83 (NR)	105 (34.2%)	222	Mixed	Mixed	DSM-IV	CAPS: 73.74 (16.7)	Paroxetine (20–50) versus placebo	12
Tucker et al., 2003	25/23	39.15 (12.21)	13 (27.08%)	41	Mixed	Mixed	DSM-IV	CAPS: 87.6 (14.48)	Citalopram 20–50 versus sertraline 50–200	10
Tucker et al., 2003	25/10	38.51 (11.12)	10 (28.57%)	29	Mixed	Mixed	DSM-IV	CAPS: 91.91 (10.9)	Citalopram 20–50 versus placebo	10
Tucker et al., 2003	23/10	38.4 (11.43)	7 (21.21%)	31	Mixed	Mixed	DSM-IV	CAPS: 87.03 (16.38)	Sertraline 50–200 versus placebo	10
Tucker et al., 2007	19/19	41.5 (10.47)	8 (21.05)	34	Civilians	Mixed	DSM-IV	CAPS: 89.7 (13.64)	Topiramate (25–400) versus placebo	12
van der Kolk et al., 1994	33/31	40.36 (7.07)	42 (65.63%)	NR	Mixed	Mixed	DSM-III-R	NR	Fluoxetine 20–60 mg or placebo	5
van der Kolk et al., 2007	30/29	34.89 (12.81)	8 (13.6%)	39	Mixed	Mixed	DSM-IV	CAPS: 72.03 (13.2)	Fluoxetine 30 (10–60) versus placebo	8
Villarreal et al., 2016	42/38	52.95 (11.07)	75 (93.75%)	42	Veterans	All combat-related	DSM-IV	CAPS: 73.12 (14.24)	Quetiapine (25–800) versus placebo	12
Yeh et al., 2011	17/14	40.45 (11.71)	10 (32.26%)	NR	Civilians	Violence-related	DSM-IV	CAPS: 73.06 (18.65)	Topiramate102.94 (25–200) versus placebo	12
Zohar et al., 2002	23/19	39.64 (7.56)	37 (88%)	NR	Veterans	32 combat-related	DSM-III-R	CAPS: 92.15 (12.5)	Sertraline 50–200 mg/d versus placebo	10

CAPS, Clinician-Administered PTSD Scale; DSM-IV-TR, the Diagnostic and Statistical Manual of Mental Disorders; ED 4, Text Rev; DTS, Davidson Trauma Scale; IES-R, the Impact of Event Scale-Revised; M.I.N.I., the Mini-International Neuropsychiatric Interview; NR, not reported; PTSD, post-traumatic stress disorder; PCL-M, the Patient Checklist for PTSD-Military Version; SI-PTSD, Structured Interview for Post-traumatic Stress Disorder; SPRINT, Short Post Traumatic Stress Disorder Rating Interview.

### Quality Assessment

The results of the quality assessment are shown in **Supplemental Table 3**. The following studies were judged to have a low risk of bias: 22 studies with sufficient information on the generation of appropriate random sequences; 18 studies with a full description of the allocation concealment; and 29 studies with sufficient information on the blinding of outcome assessment.

### Outcomes for Active Drug vs. Placebo

**Supplemental Table 4** shows the results of analysis of active drugs and placebo for different classification of pharmacological mechanisms and specific drug branches. **Supplemental Table 5** shows the results of the stratification analysis. Overall, active drugs could significantly reduce the PTSD total severity, symptoms of re-experiencing, avoidance, hyperarousal, depression, and anxiety relative to other active-comparators.

### Efficacy

#### Change in Total PTSD Symptoms Based on a Clinician-Assessed Scale

Compared with placebo, all active drugs (SMD, −0.33; 95% CI, −0.43 to −0.23), different drug classifications like atypical antipsychotics (SMD = −0.30, 95% CI: −0.46 to −0.13), SNRIs (SMD = −0.29, 95% CI: −0.44 to −0.14), SSRIs (SMD = −0.33, 95% CI: −0.40 to −0.25), and TeCAs (SMD = −1.87, 95% CI: −2.58 to −0.89) had significant efficacy in the change of clinician-assessed scale (**Supplemental Table 4**). However, the following specific drug branches presented more beneficial effects than placebo (**Supplemental Table 4**): quetiapine (SMD = −0.49, 95% CI: −0.93 to −0.04), risperidone (SMD = −0.23, 95% CI: −0.42 to −0.03), fluoxetine (SMD = −0.27, 95% CI: −0.42 to −0.12), hydroxyzine (SMD = −1.56, 95% CI: −2.11 to −1.02), mirtazapine (SMD = −1.87, 95% CI: −2.85 to −0.89), olanzapine (SMD = −0.66, 95% CI: −1.19 to −0.13), paroxetine (SMD = −0.48, 95% CI: −0.60 to −0.36), sertraline (SMD = −0.22, 95% CI: −0.35 to −0.10), and venlafaxine (SMD = −0.29, 95% CI: −0.44 to −0.14).

#### Symptoms of Re-Experiencing

Compared with placebo, all active drugs (SMD = −0.32, 95% CI: −0.41 to −0.23) and different drug classifications, such as atypical antipsychotics (SMD = −0.37, 95% CI: −0.54 to −0.19), MAOI (SMD = −1.07, 95% CI: −1.77 to −0.38), SNRIs (SMD = −0.20, 95% CI: −0.35 to −0.05), and SSRIs (SMD = −0.28, 95% CI: −0.36 to −0.20), significantly reduced the symptoms of re-experiencing (**Supplemental Table 4**). For specific drug branches, amitriptyline (SMD = −0.75, 95% CI: −1.46 to −0.04), quetiapine (SMD = −0.53, 95% CI: −0.98 to −0.09), risperidone (SMD = −0.36, 95% CI: −0.56 to −0.15), fluoxetine (SMD = −0.24, 95% CI: −0.41 to −0.08), paroxetine (SMD = −0.39, 95% CI: −0.51 to −0.27), sertraline (SMD = −0.26, 95% CI: −0.47 to −0.06), phenelzine (SMD = −1.07, 95% CI: −1.77 to −0.38), and venlafaxine (SMD = −0.20, 95% CI: −0.35 to −0.05) demonstrated greater efficacy than placebo (**Supplemental Table 4**).

#### Symptoms of Avoidance

Compared with placebo, all active drugs (SMD = −0.27, 95% CI: −0.33 to −0.21) and different drug classifications, such as MAOIs (SMD = −0.81, 95% CI: −1.49 to −0.14), SNRIs (SMD=−0.28, 95% CI: −0.43 to −0.13), and SSRIs (SMD = −0.34, 95% CI: −0.41 to −0.26), significantly reduced the symptoms of avoidance (**Supplemental Table 4**). By contrast, in the specific drug branches, amitriptyline (SMD = −0.90, 95% CI:−1.62 to −0.18), phenelzine (SMD = −0.81, 95% CI: −1.49 to −0.14), venlafaxine (SMD = −0.28, 95% CI:−0.43 to −0.13), fluoxetine (SMD = −0.20, 95% CI: −0.37 to −0.04), sertraline (SMD = −0.33, 95% CI: −0.46 to −0.20), and paroxetine (SMD = −0.42, 95% CI: −0.54 to −0.30) were associated with generally greater improvement for relieving avoidance symptoms (**Supplemental Table 4**).

#### Symptoms of Hyperarousal

Compared with placebo, all active drugs (SMD = −0.34, 95% CI: −0.34 to −0.22) and different drug classifications, such as atypical antipsychotics (SMD = −0.37, 95% CI: −0.54 to −0.20), SNRIs (SMD = −0.25, 95% CI: −0.40 to −0.10), and SSRIs (SMD = −0.37, 95% CI: −0.49 to −0.25), significantly reduced the severity of hyperarousal symptoms (**Supplemental Table 4**). For specific drug branches, quetiapine (SMD = −0.55, 95% CI: −1.00 to −0.10), fluoxetine (SMD = −0.27, 95% CI: −0.43 to −0.10), paroxetine (SMD = −0.40, 95% CI: −0.52 to −0.27), sertraline (SMD = −0.45, 95% CI: −0.75 to −0.16), and venlafaxine (SMD = −0.25, 95% CI: −0.40 to −0.10), significantly reduced the severity of hyperarousal (**Supplemental Table 4**).

#### Symptoms of Depression

Compared with placebo, depression symptoms were observed to have been noticeably reduced by using all active drugs (SMD = −0.28, 95% CI: −0.34 to −0.23) and different drug classifications, such as alpha blockers (SMD = −0.54, 95% CI: −0.95 to −0.13), atypical antipsychotics (SMD = −0.33, 95% CI: −0.51 to −0.15), SNRIs (SMD = −0.21, 95% CI: −0.36 to −0.06), SSRIs (SMD = −0.28, 95% CI: −0.41 to −0.16), and TeCAs (SMD = −0.91, 95% CI: −1.76 to −0.05), in a depression-related rating scale (**Supplemental Table 4**). For specific drug branches, prazosin (SMD = −0.54, 95% CI: −0.95 to −0.13), amitriptyline (SMD = −1.16, 95% CI: −1.90 to −0.41), olanzapine (SMD = −0.81, 95% CI: −1.41 to −0.20), quetiapine (SMD = −0.63, 95% CI: −1.08 to −0.18), mirtazapine (SMD = −0.91, 95% CI: −1.76 to −0.05), vilazodone (SMD = −0.21, 95% CI: −0.36 to −0.06), fluoxetine (SMD = −0.25, 95% CI: −0.40 to −0.10), and paroxetine (SMD = −0.49, 95% CI: −0.61 to −0.36) significantly relieved the symptoms of depression (**Supplemental Table 4**).

#### Symptoms of Anxiety

Compared with placebo, all active drugs (SMD = −0.23, 95% CI: −0.33 to −0.14) and different drug classifications, such as atypical antipsychotics (SMD = −0.32, 95% CI: −0.51 to −0.12), MAOI (SMD = −0.67, 95% CI: −1.34 to −0.01), TCAs (SMD = −0.68, 95% CI: −1.16 to −0.21), and TeCAs (SMD = −0.89, 95% CI: −1.74 to −0.04), significantly reduced the symptoms of anxiety (**Supplemental Table 4**). For specific drug branches, amitriptyline (SMD = −0.99, 95% CI:−1.72 to −0.26), fluoxetine (SMD = −0.28, 95% CI: −0.44 to −0.12, I^2^ = 0%), mirtazapine (SMD = −0.89, 95% CI: −1.74 to −0.04), and phenelzine (SMD = −0.67, 95% CI: −1.34 to −0.01) significantly reduced the symptoms of anxiety (**Supplemental Table 4**).

### Acceptability

#### All-Cause Discontinuation Rate

Compared with placebo, all different drug classifications and specific drug branches, except for phenelzine (RR = 0.32, 95% CI: 0.12 to 0.80), did not show statistically significant differences in the all-cause discontinuation rate (**Supplemental Table 4**).

#### Discontinuation Rate Due to Adverse Effects

In terms of discontinuation rate due to adverse effects, all active drugs (RR = 1.47, 95% CI: 1.24 to 1.75), different drug classifications like atypical antipsychotics (RR = 2.06, 95% CI: 1.10 to 3.84) and SSRIs (RR = 1.37, 95% CI: 1.08 to 1.74), and specific drug branches, which included only paroxetine (RR = 1.45, 95% CI: 1.02 to 2.07) and topiramate (RR = 2.61, 95% CI: 1.09 to 6.25), had significantly higher discontinuation rate due to adverse effects than placebo (**Supplemental Table 4**).

### Outcomes for Active Drug vs. Active Comparators

We also synthesised active-comparators studies separately to assess the differences between active drugs and active comparators (**Supplemental Table 5**). The analysis showed that sertraline relative to citalopram significantly reduces total severity of PTSD based on a clinician-assessed scale (SMD = 0.65, 95% CI: 0.07 to 1.23) and avoidance symptoms (SMD = 0.93, 95% CI: 0.33 to 1.52). For symptoms of re-experiencing, the different drug classifications, including MAOI vs. TCAs (SMD = −0.64, 95% CI: −1.27 to −0.02), and specific drug branches, including phenelzine vs imipramine (SMD = −0.64, 95% CI: −1.27 to −0.02), had statistically significant differences.

### Stratified Analyses

#### Severity of Trauma Based on CAPS Scores

**Figure 2:**
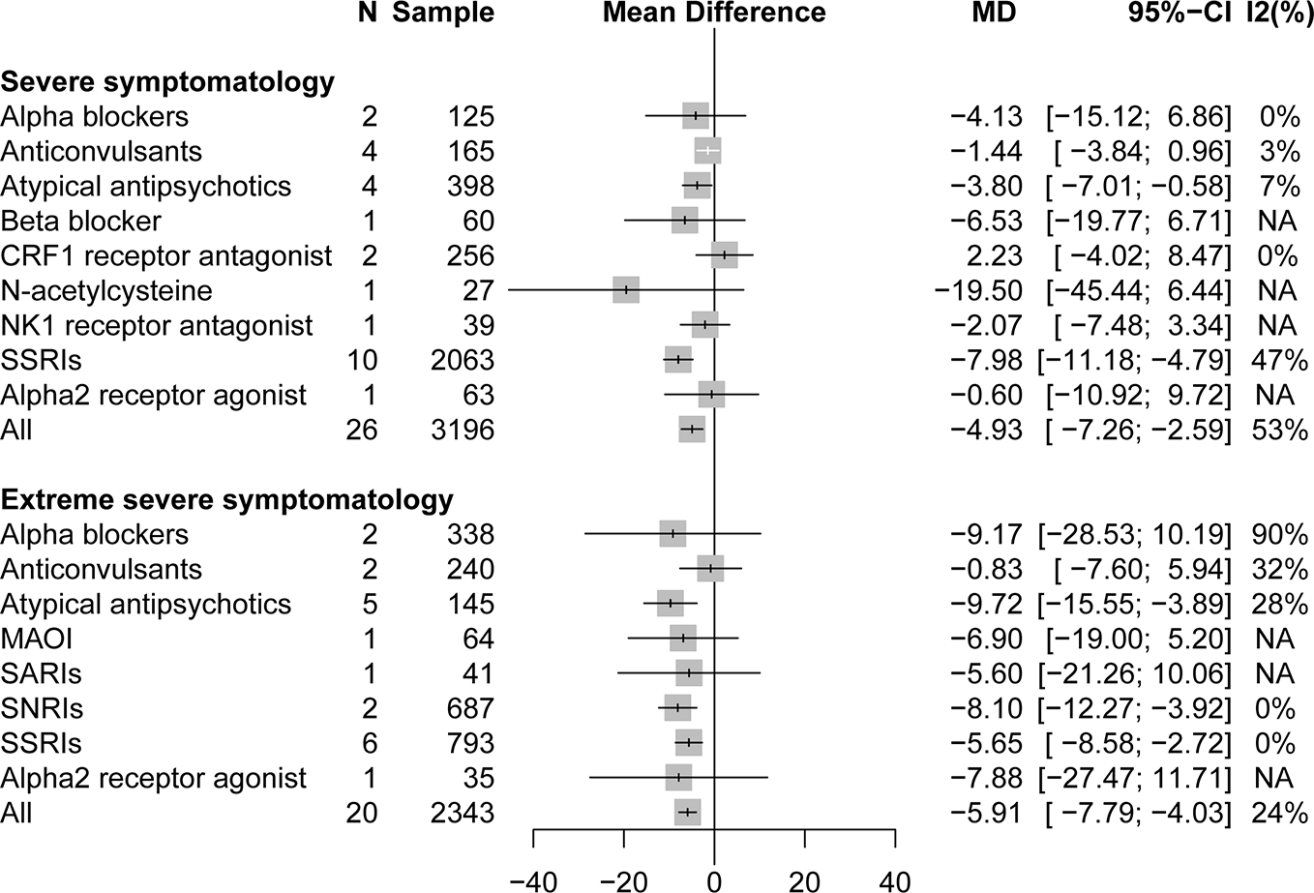
Forest plot based on stratified analyses of severity of trauma based on CAPS scores of placebo-controlled comparisons. MAOIs, monoamine oxidase inhibitors; NK1, neurokinin-1 receptor antagonist; SARIs, serotonin antagonist and reuptake inhibitors; SNRIs, serotonin and norepinephrine reuptake inhibitors; SSRIs, selective serotonin reuptake inhibitors; NA, not applicable.

**Figure 3:**
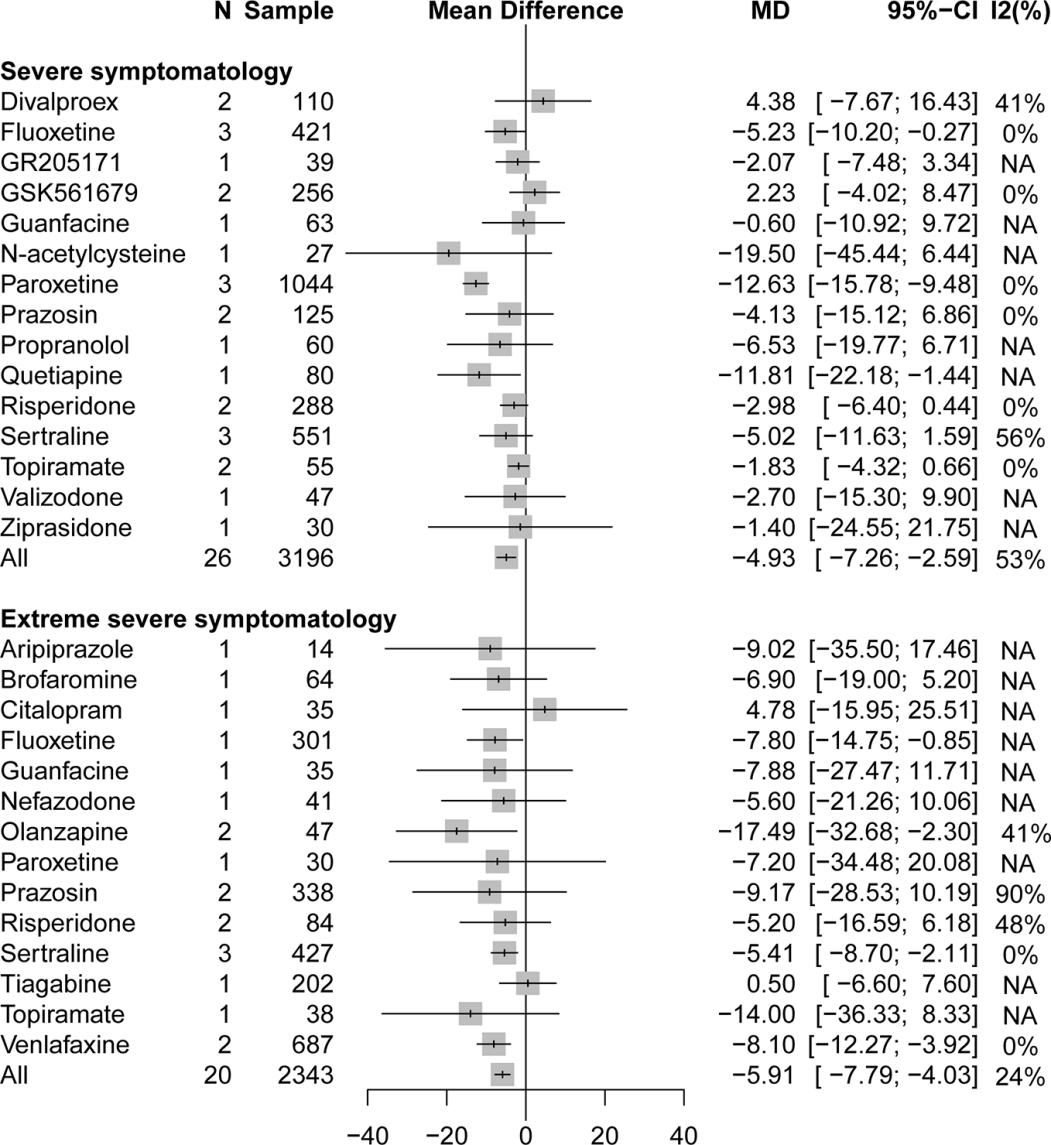
Forest plot based on stratified analyses of severity of trauma based on CAPS scores of placebo-controlled comparisons in specific drug branches.

A stratified analysis of drug treatment in population with severe PTSD symptomatology (CAPS score of 60 to 79) showed that compared with placebo, all active drugs (MD = −4.93, 95% CI: −7.27 to −2.59) and different drug classifications, such as atypical antipsychotics (MD = −3.80, 95% CI: −7.01 to −0.58) and SSRIs (MD = −7.98, 95% CI: −11.18 to −4.79), can significantly reduce the severity of PTSD total symptoms in **Figure 2**. Compared with placebo, in specific drug branches, fluoxetine (MD = −5.23, 95% CI: −10.20 to −0.27), paroxetine (MD = −12.63, 95% CI: −15.78 to −9.48), and quetiapine (MD = −11.81, 95% CI: −22.18 to −1.44) can significantly reduce the CAPS total score in patients with severe PTSD symptomatology (**Figure 3**). For patients with extreme PTSD symptomatology (CAPS ≥80), all active drugs (SMD = −5.91, 95% CI: −7.79 to −4.03) and different drug classifications, such as atypical antipsychotics (MD = −9.72, 95% CI: −15.55 to −3.89), SNRI (MD = −8.10, 95% CI: −12.27 to −3.92), and SSRIs (MD = −5.65, 95% CI: −8.58 to −2.72), can significantly reduce the CAPS total score relative to placebo in **Figure 2**. In specific drug branches, fluoxetine (MD = −7.80, 95% CI: −14.75 to −0.85), olanzapine (MD = −17.49, 95% CI: −32.68 to −2.30), sertraline (MD = −5.41, 95% CI: −8.70 to −2.11), and venlafaxine (MD = −8.10, 95% CI: −12.27 to −3.92) can significantly reduce the CAPS total score relative to placebo in **Figure 3**.

#### Different Populations

**Figure 4:**
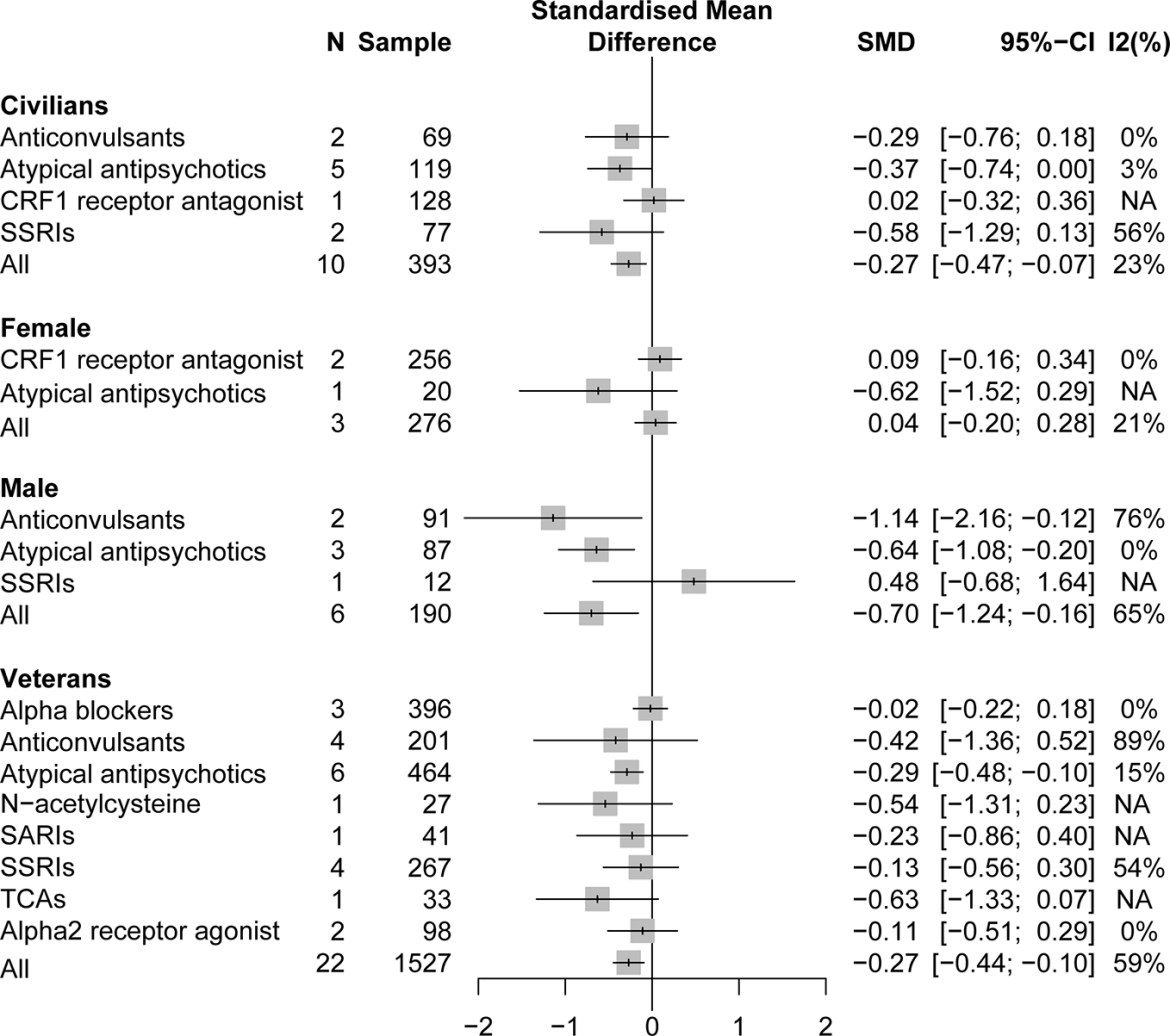
Forest plot based on stratified analyses of different populations and gender of placebo-controlled comparisons. TCAs, tricyclic antidepressants.

**Figure 5:**
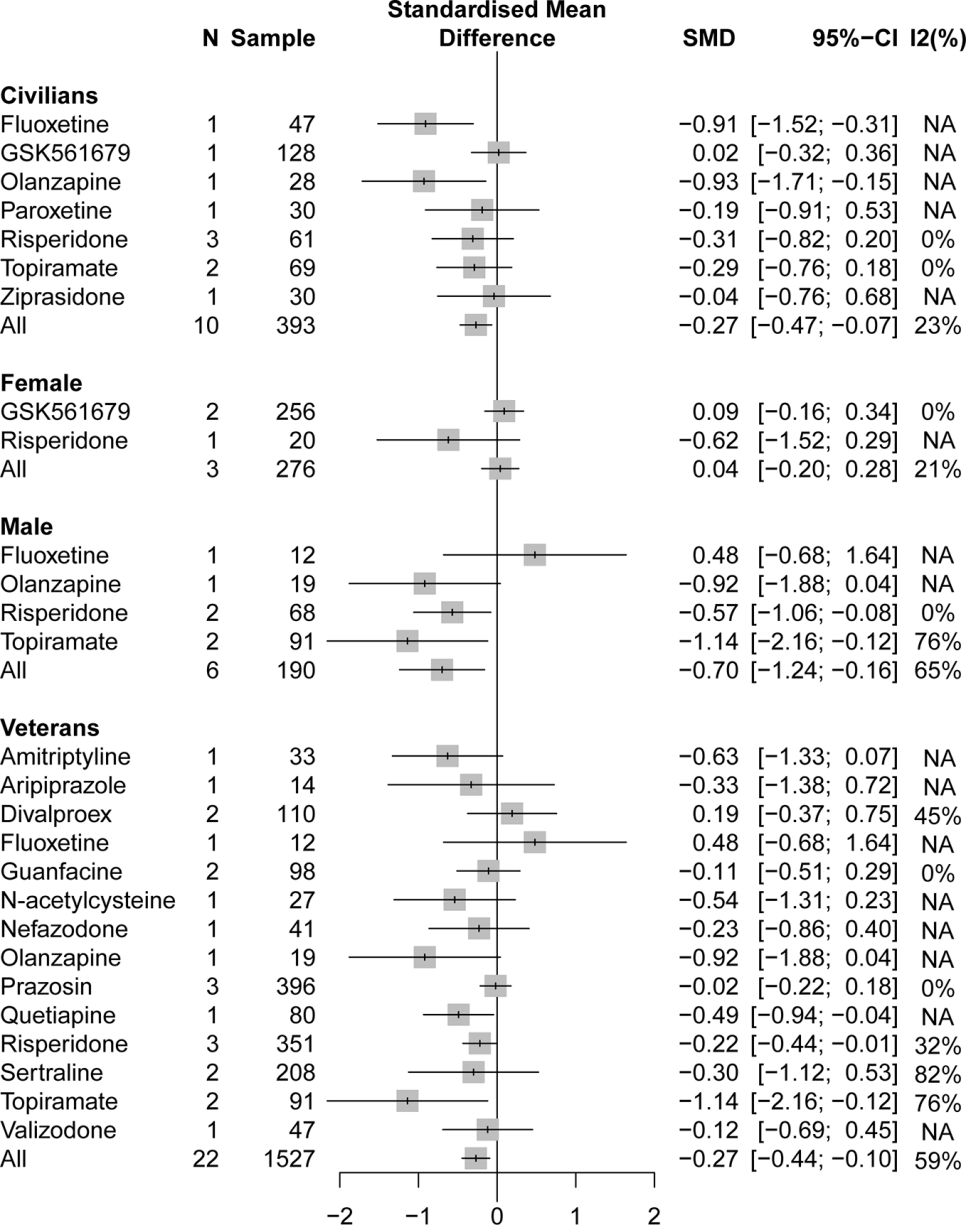
Forest plot based on stratified analyses of different populations and gender of placebo-controlled comparisons in specific drug branch.

A pooling analysis of the different drug classifications compared with placebo for veterans indicated that all active drugs (SMD = −0.27, 95% CI: −0.44 to −0.10) and atypical antipsychotics (SMD = −0.29, 95% CI: −0.48 to −0.10) can significantly reduce the clinician-assessed scale, as can be observed in **Figure 4**. In specific drug branches, quetiapine (SMD = −0.49, 95% CI: −0.93 to −0.04), risperidone (SMD = −0.22, 95% CI: −0.44 to −0.01), and topiramate (SMD = −1.14, 95% CI: −2.16 to −0.12) can significantly reduce the total PTSD severity based on a clinician-assessed scale in **Figure 4**. For civilian PTSD patients, compared with placebo, all active drugs (SMD = −0.27, 95% CI: −0.47 to −0.07), olanzapine (SMD = −0.93, 95% CI: −1.71 to −0.15), and fluoxetine (SMD = −0.91, 95% CI: −1.52 to −0.31) had a significant efficacy in **Figure 5**.

#### Gender

A pooling analysis of studies involving all-male population showed that all active drugs (SMD = −0.70, 95% CI: −1.24 to −0.16), risperidone (SMD = −0.57, 95% CI: −1.06 to −0.08), and topiramate (SMD = −1.14, 95% CI: −2.16 to −0.12) can significantly reduce a clinician-assessed scale relative to placebo in **Figures 4** and **5**. However, the results of the combined analysis of female patients indicated that no drug has a better effect than placebo.

#### Age and Races

We did not find any older populations (over 60 years of age) involved in our study, and there is no evidence of a comparison between older and non-older adults. For the races, only one study was concerned on a single race. A stratification analysis based on racially diverse populations has not been performed in current studies.

#### Therapeutic Regimens

Compared active drugs with placebo, the monotherapy demonstrated a significant reduction in CAPS scores (SMD = −0.31, 95% CI: −0.41 to −0.21, I < sp > 2 = 63%), meanwhile, the adjunctive therapy also indicated a positive effect on reducing CAPS scores (SMD = −0.49, 95% CI: −0.92 to −0.06, I^2^ = 63%).

### Meta-Regression

For the change in overall PTSD symptoms, the meta-regression results for patients’ age resulted in a significant coefficient (coefficient = 0.02, P = 0.046), implying that treatments, compared with placebo, in younger patients tend to be more effective than in older patients. A meta-regression model of the severity of trauma showed that active treatments, compared with placebo, tend to be less effective in studies with more severe patients (coefficient = 0.03, P = 0.75). Meanwhile, the meta-regression of publication year (P = 0.26) and sponsorship (P = 0.68) indicated that it had not reached statistical difference.

### Publication Bias

The results of the pooled analysis were not reversed by the trim-and-fill method for the study of anticonvulsants and SSRIs. However, in terms of discontinuation rate due to adverse effects for atypical antipsychotics compared with placebo, the results of the pooled analysis were reversed (RR = 1.57, 95% CI: 0.86 to 2.87), suggesting that publication bias may affect the robustness of the findings of atypical antipsychotics (**Supplemental Table 6**).

## Discussion

Based on 78 RCTs from 66 studies, this study was the largest and latest meta-analysis of pharmacological treatments for PTSD in adults. We obtained a more comprehensive evidence for a detailed search of published literature; from the recent meta-analysis (Cipriani et al., 2018), this meta-analysis covered 15 additional studies. We investigated important results related to changes in PTSD total symptoms, reduction rate of core and other symptoms, all-cause discontinuation, discontinuation due to adverse effects, which were chosen to estimate pharmacological treatments efficacy and acceptability. Necessary considerations to this study were the extrapolation of evidences based on specific treatment options for PTSD and exploration of the applicability of key evidences for the different individual patient levels and sub-symptoms, such as basic characteristics of population, severity of trauma, special population, different classifications of pharmacological mechanisms, and specific drug branches.

The APA and NICE guidelines (Association, 2017; Excellence NIfHaC, 2018) indicate that fluoxetine, paroxetine, sertraline, and venlafaxine should be recommended for PTSD drug therapy. The results of our meta-analysis are consistent with this recommendation and provide more reliable evidence for these drugs. After conducting a stratification analysis and considering fully the modification factors, we clearly defined the scope of application for these four drugs. Our study showed that paroxetine can be used for severe PTSD symptom levels, whereas sertraline and venlafaxine can be used for extremely severe PTSD symptom levels. Meanwhile, fluoxetine can be used for the treatment of severe or extremely severe PTSD symptoms, especially for civilian-related trauma. For different doses, this study found that the discontinuation rate due to adverse effects of 40 mg/day of fluoxetine is significantly higher than 20 mg/day of fluoxetine; however, there was no significant difference in efficacy, including reduction of various symptoms (Martenyi et al., 2007). By contrast, the use of different doses (20 mg/day vs. 40 mg/day) of paroxetine did not show significant differences in efficacy and acceptability (Marshall et al., 2001). Unfortunately, this study found no evidence in the comparisons of different doses within venlafaxine and sertraline. For the comparison between venlafaxine and sertraline, there was also no significant difference, but no evidence was found for pair-wise comparisons of other two drugs.

The APA guidelines also indicate that there is currently insufficient evidence to confirm the efficacy of risperidone and topiramate. However, the risperidone and topiramate showed a significant effect when compared with placebo in our meta-analysis. This study demonstrated a potential tendency that risperidone and topiramate could be considered in male patients with PTSD, whereas topiramate is more suitable for male veterans with PTSD. Risperidone was as an adjunctive drug in five of the six studies on risperidone (Hamner et al., 2003; Reich et al., 2004; Bartzokis et al., 2005; Padala et al., 2006; Rothbaum et al., 2008; Krystal et al., 2011); only one study (Padala et al., 2006) involved a monotherapy drug. It is common for PTSD patients to be combined with other psychiatric disorders that rely on medicine interventions. Therefore, we evaluated the effect of the monotherapy and adjunctive drugs. The results in this study suggested either monotherapy or adjunctive therapy could decrease CAPS scores, which were basically expected. Our subgroup analysis found that risperidone had a more significant efficacy as a monotherapy drug than as an adjunctive drug. Of the six studies on topiramate, only Akuchekian’s (Akuchekian et al., 2004) from 2004 results indicated higher effect values; the other studies found no significant statistical significance. The heterogeneity of the pooling analysis of the efficacy of topiramate may be attributed to the difference in results. The maximum dose of 500 mg/day of titrated topiramate in Akuchekian’s study (Akuchekian et al., 2004) was higher than the 400 mg/day in other studies; the population in Akuchekian’s study (Akuchekian et al., 2004) also had a comorbid psychotic illness while having a high duration of illness (mean duration of 17 years). In the future, the study of topiramate must consider the difference in drug efficacy caused by higher doses and different populations.

The NICE guideline (Excellence NIfHaC, 2018) also pointed out the potential role of atypical antipsychotics for the treatment of PTSD. A study (Berger et al., 2007) showed that atypical antipsychotics may be an alternative when PTSD does not respond to SSRIs. Veterans with comorbidity of PTSD (McCauley et al., 2012) are more difficult to treat than non-veterans patients; they have higher suicide attempts and worse treatment adherence (McCauley et al., 2012; Reisman, 2016). Therefore, compared with SSRIs, atypical antipsychotics may be more considered for veterans as a special group. Our research results showed that atypical antipsychotics are effective in the treatment of PTSD total symptoms, especially for veterans. This finding is different from the recent a meta-analysis (Hoskins et al., 2015) of pharmaceutical PTSD. We found that quetiapine and topiramate significantly reduce the severity of PTSD for veterans compared with placebo in all active drugs. Moreover, olanzapine is suitable for civilian trauma and extremely severe PTSD symptoms.

Our study demonstrated that paroxetine, sertraline, venlafaxine, and fluoxetine can also be used as the first choice of treatment for the three main core symptoms. A pooling analysis of atypical antipsychotics drugs involved a moderate heterogeneity (40%) for avoidance symptoms, and subgroup analysis showed heterogeneity in two studies (Butterfield et al., 2001; Carey et al., 2012) of olanzapine; excluding these latter two studies, atypical antipsychotics drugs did not show significant efficacy. Risperidone can also reduce re-experiencing symptoms and achieve significant efficacy. Of the studies on risperidone, only Krystal’s study (Krystal et al., 2011) used a large sample, showing a significant advantage in reducing hyperarousal and re-experiencing symptoms. For patients diagnosed with military-related PTSD and had antidepressant-resistant symptoms, risperidone did not significantly reduce the overall PTSD symptoms. Further researches are required to confirm or revise this result. Prazosin, paroxetine, fluoxetine, and venlafaxine can be considered a priority in reducing symptoms of depression drug. Sertraline did not show a significant advantage in reducing depression symptoms. Surprisingly, the pooling analysis of SSRIs did not show an advantage in decreasing the symptoms of anxiety. A further subgroup analysis showed that fluoxetine significantly reduces the symptoms of anxiety.

Compared with previous meta-analyses, we conducted a specific stratified analysis of age, gender, patient type, and severity of PTSD for the maximized extrapolation of evidence based on the current guideline (Association, 2017). Unfortunately, only one RCT (Li et al., 2017) recruited a population of a single race (Asian). There was also no trial of PTSD for older adults (over 60 years of age) in these studies. Therefore, our conclusions may only be suitable for non-older adults (18–60 years old). Additionally, the meta-regression analysis found that the efficacy of a drug becomes less with increasing age. Relevant studies (Olff, 2017) showed that compared with men, the risk of suffering from PTSD was twice or thrice higher for women, which may be related to psychosocial and biological characteristics. Female patients were only based on three RCTs that analysed GSK561679 and risperidone. The results showed that there were currently no effective drugs for female PTSD patients, which could be attributed to the small sample size. Consequently, more studies with a large sample are needed to verify this finding.

Although these recommended drugs based on a comparative analysis of placebo-controlled trials showed significant advantages over placebo in terms of efficacy, the effect size of these drugs almost even did not reach the moderate effect (SMD range of 0.5–0.8) according to Cohen’s criteria (Cohen, 2013). On the contrary, those drugs (e.g., hydroxyzine, mirtazapine, and phenelzine) that achieved large effect values were derived from small sample studies. Most studies compare active drugs with placebo. We also focused on active-comparators studies to explore the differences among drugs; unfortunately, the information obtained was quite small. This finding is consistent with the conclusion of a previous meta-analysis (Cipriani et al., 2018). Phenelzine is the only drug that was found to be significantly better than the placebo in terms of all-cause discontinuation rate. The effect value of the all-cause discontinuation for another drug group after subgroup analysis was around 1. Compared with placebo, atypical antipsychotics and SSRIs had significantly higher discontinuation due to adverse effects. The discontinuation due to adverse drug reactions was significantly higher than that of placebo by approximately 1.47 times. Specifically, when using paroxetine and topiramate clinically, the adverse reactions were significantly higher than placebo. We found that the discontinuation due to adverse effects was attributed to CNS, including impaired cognition, paraesthesia, headache, dizziness or light-headedness, sedation, and sexual dysfunction, by analysing the original study on topiramate. The main cause of adverse reactions during a headache was the reason for discontinuation of paroxetine. Higher discontinuation due to adverse effects was observed in sertraline compared with placebo but was not significant.

A network meta-analysis that compares multiple treatments is clinically more useful than that an analysis using pairwise comparisons alone because, in the right circumstances, the former enables competing interventions to be ranked (Cipriani et al., 2013). However, considering the small amount of available data, the results of a published network meta-analysis (Cipriani et al., 2018) of pharmacotherapy for PTSD in 2018 are probably not robust enough to suggest that it is the best option as a drug of choice. Most importantly, this network meta-analysis did not consider modifiers for effect of results at the individual patient level (e.g., age, sex, severity of symptoms, special population, or type of trauma). Cipriani’s study (Cipriani et al., 2018) showed that phenelzine has a huge advantage compared with other drugs based on the indirect comparisons of active-comparators experiments. However, the efficacy of phenelzine was only derived from one small RCT (Kosten et al., 1991) with 60 participants in 1991. Therefore, the accuracy of phenelzine requires more research to confirm.

Based on current guidelines and evidence, we can make the following recommendations for clinicians and future research. The core symptoms of PTSD should be treated preferentially (Excellence NIfHaC, 2018) because the symptoms of comorbidity usually improve with the success of treatment of PTSD. However, comorbidity symptoms must be treated first if they seriously impact a patient’s life or are sufficient to make the treatment of PTSD difficult. An example is the comorbidity of suicide and depression of veterans (Reisman, 2016). The evidence for the drugs recommended in the current guidelines(Association, 2017; Excellence NIfHaC, 2018) has been updated in our study, but the efficacy of these drugs is still quite small (around 0.3). These drugs may be more suitable to relieve certain emergency symptoms in cases where psychotherapy does not yield rapid efficacy. The clinician should consider fully the individual factors for the choice of drugs rather than fixed use of a drug to treat, such as the severity of symptom, different populations, or some critical sub-symptoms. Clinicians may use our results to find a preliminary drug selection strategy and should consider a patient’s own preferences (A. McHugh et al., 2013; Zoellner et al., 2019). Especially for veterans, common drugs may not be suitable for patients who are resistant to common psychotropic substances, such as SSRIs. Atypical antipsychotics can be used as a strategy for clinical application (Berger et al., 2007). In the future, making great progress in pharmacological treatments of PTSD may need to be researched for some special drugs that have strong effect values from small sample studies. Future research should continue to conduct rigorous stratification analysis for different populations or baseline symptoms to determine individual differences among drugs. Differences in efficacy due to dose variances are worthy of further investigation in the same drug. Our research has some limitations. First, most studies have focused on placebo-controlled trials rather than on active-comparators trials, making it difficult to obtain direct evidence of benefit. After the subgroup analysis to eliminate clinical heterogeneity as much as possible was strictly implemented, we used I^2^ to select fixed or random effect models based on the Cochrane’s Handbook(Higgins and Green, 2011) for deal with statistical heterogeneity. It is worth noting that the uncertain pooled results of small sample studies, such as phenelzine, may affect our estimation of the true effect size no matter which effect model is implemented (Borenstein et al., 2010; Riley et al., 2011). We look forward to more large samples and high-quality studies to confirm our findings in the future. Second, up to two thirds of the research included in the original study was supported by funds from pharmaceutical companies, which may have some impact on our conclusions. Third, most RCTs included in our analysis did not report adequate information on random sequence generation and allocation concealment. The insufficient description of study design may lead to bias in the results.

Pharmacological interventions can be effective in PTSD, which may reduce its core symptoms (re-experiencing, avoidance, and hyperarousal) and thus should be considered in improving the symptoms of PTSD. This systematic review, meta-analysis supports the use of SSRIs, SNRIs, and atypical antipsychotics as pharmacological interventions for overall symptoms of PTSD and three core symptoms, except for atypical antipsychotics for the improvement of avoidance symptoms. Different drugs should be selected for the treatment of patients with varying clinical features of PTSD. The atypical antipsychotics should be prioritized rather than SSRIs for veterans and males. SSRIs, SNRIs, and atypical antipsychotics are the medication of choice for patients with severe or extremely severe PTSD symptoms. There is insufficient evidence for drug selection for PTSD treatment in civilians and females patients.

The magnitude of pharmacological interventions for PTSD is small and evidence regarding the efficacy for PTSD is inadequate, thereby highlighting the need for more research in this area to confirm the use of pharmacological treatments for this disorder. Despite these objective limitations, our findings represented the best currently available evidence to guide the initial choice of pharmacological treatments of PTSD in adults and inform future guidelines in deciding what drugs to use to treat PTSD.

## Author Contributions

Substantial contributions to conception and design or analysis and interpretation of data: Z-DH, JL, Y-FZ, SL, H-YG, L-LL, Z-YY, Y-MN, and CZ. Substantial contributions to drafting the article or revising it critically for important intellectual content: Z-DH, JL, and Y-FZ. Final approval of the version to be published: Z-DH, JL, Y-FZ, SL, H-YG, L-LL, Z-YY, Y-MN, and CZ.

## Conflict of Interest

The authors declare that the research was conducted in the absence of any commercial or financial relationships that could be construed as a potential conflict of interest.
