# Supplementary material for: Comparative Efficacy and Acceptability of Pharmaceutical Management for Adults With Post-Traumatic Stress Disorder: A Systematic Review and Meta-Analysis
Source: Front Pharmacol. 2020 May 8;11:559. doi: 10.3389/fphar.2020.00559 (PMC7225303; doi:10.3389/fphar.2020.00559)
Supplement: Supplementary file 1 [file DataSheet_1.docx]

**Electronic Supplementary Material**

**Supplemental Method 1** Search strategy

**Supplemental Table 1** The classification of drugs, abbreviation and pharmacological agents used in treating post-traumatic stress disorder

**Supplemental Table 2** Funding source of included studies

**Supplemental Table 3** Risk of bais for included studies

**Supplemental Table 4** Placebo-controlled comparisons for efficacy and acceptability of pharmacological management for PTSD

**Note:** MAOIs: Monoamine oxidase inhibitors, NDRIs: Norepinephrine and dopamine reuptake inhibitors, NK1: Neurokinin-1 receptor antagonist, NRI: Norepinephrine reuptake inhibitor, SARIs: Serotonin antagonist and reuptake inhibitors, SMSs: Serotonin modulator and stimulators, SNRIs: Serotonin and norepinephrine reuptake inhibitors, SSRIs: Selective serotonin reuptake inhibitors, TCAs: Tricyclic antidepressants, TeCAs: Tetracyclic antidepressants, NA: Not applicable.

**Supplemental Table 5** Active comparisons for efficacy and acceptability of pharmacological management for PTSD

**Note:** MAOIs: Monoamine oxidase inhibitors, NRI: Norepinephrine reuptake inhibitor, SNRIs: Serotonin and norepinephrine reuptake inhibitors, SSRIs: Selective serotonin reuptake inhibitors, TCAs: Tricyclic antidepressants, NA: Not applicable.

**Supplemental Table 6** Publication bias using trim-and-fill method of placebo-controlled comparisons for efficacy and acceptability of pharmacological management in all outcomes

**Supplemental Method 1** Search strategy

**1. Ovid MEDLINE(R) and Epub Ahead of Print, In-Process & Other Non-Indexed Citations, Daily, and Versions(R)** **<1946 to June 21, 2019>**

1. (Stress Disorders, Post-Traumatic).mp. /(30288)
2. (post-traumatic stress disorder).mp. /(10187)
3. (post-traumatic stress disorders).mp. /(332)
4. (disorder* AND post-traumatic).ti,ab. /(11396)
5. (Stress Disorders, Traumatic).mp. /(1088)
6. (Combat Disorders).mp. /(3064)
7. PTSD.mp. /(202478)
8. #1 OR #2 OR #3 OR #4 OR #5 OR #6 OR #7 /(41680)
9. Limit 8 to (“all adult (19 plus years)” or “young adult (19 to 24 years)” or “adult (19 to 44 years)” or “young adult and adult (19-24 and 19-44)” or “middle age (45 to 64 years)” or “middle aged (45 plus years)” or “all aged (65 and over)” or “aged (80 and over)”) /(21481)
10. Benzodiazepines.mp. /(32300)
11. (Antidepressive Agents, Tricyclic).mp. /(10225)
12. Anticonvulsants.mp. /(53099)
13. (Adrenergic alpha-Antagonists).mp. /(14230)
14. (Antipsychotic Agents).mp. /(52622)
15. (Antidepressive Agents).mp. /(56523)
16. (citalopram OR escitalopram OR fluoxetine OR fluvoxamine OR paroxetine OR sertraline OR desvenlafaxine OR venlafaxine OR duloxetine OR imipramine OR amitriptyline OR desipramine OR bupropion OR mirtazapine OR nefazodone OR trazodone OR prazosin OR olanzapine OR risperidone OR benzodiazepines OR alprazolam OR diazepam OR lorazepam OR clonazepam OR topiramate OR tiagabine OR lamotrigine OR carbamazepine OR divalproex).mp. /(160843)
17. #10 OR #11 OR #12 OR #13 OR #14 OR #15 OR #16 /(295377)
18. #9 AND #17 /(22314)
19. Limit #18 to randomized controlled trial /(1369)

**2. EMbase+EMBase classic <1946 to June 21, 2019>**

1. exp 'stress disorders, post-traumatic' /(55385)
2. 'post-traumatic stress disorder'.mp. /(13900)
3. 'post-traumatic stress disorders'.ti,ab. /(436)
4. 'disorder* and post-traumatic'.ti,ab. /(18108)
5. exp 'stress disorders, traumatic' /(55385)
6. 'combat disorders'.mp. /(159)
7. ptsd.ti,ab. /(28393)
8. #1 OR #2 OR #3 OR #4 OR #5 OR #6 OR #7 /(61697)
9. exp 'benzodiazepines' /(214972)
10. exp 'antidepressive agents, tricyclic' /(113607)
11. exp 'adrenergic alpha-antagonists' /(311452)
12. exp 'antipsychotic agents' /(283972)
13. exp 'antidepressive agents' /(428823)
14. ('citalopram' OR 'escitalopram' OR 'fluoxetine' OR 'fluvoxamine' OR 'paroxetine' OR 'sertraline' OR 'desvenlafaxine' OR 'venlafaxine' OR 'duloxetine' OR 'imipramine' OR 'amitriptyline' OR 'desipramine' OR 'bupropion' OR 'mirtazapine' OR 'nefazodone' OR 'trazodone' OR 'prazosin' OR 'olanzapine' OR 'risperidone' OR 'benzodiazepines' OR 'alprazolam' OR 'diazepam' OR 'lorazepam' OR 'clonazepam' OR 'topiramate' OR 'tiagabine' OR 'lamotrigine' OR 'carbamazepine' OR 'divalproex').mp. /(391647)
15. #9 OR #10 OR #11 OR #12 OR #13 OR #14 /(942472)
16. #8 AND #15 /(7419)
17. Limit #16 to (randomized controlled trial and (adult <18 to 64 years> or aged <65+ years>)) /(291)

**3. CENTRAL, The** **Cochrane Library <Issue 6 of 12, June 2019>**

1. MeSH descriptor: [Stress Disorders, Post-Traumatic] explode all trees /(2175)
2. (post-traumatic stress disorder):ti,ab,kw /(2645)
3. (post-traumatic stress disorders):ti,ab,kw /(2476)
4. (disorder* AND post-traumatic):ti,ab,kw /(3243)
5. MeSH descriptor: [Stress Disorders, Traumatic] explode all trees /(2284)
6. MeSH descriptor: [Combat Disorders] explode all trees /(123)
7. (PTSD):ti,ab,kw /(3553)
8. #1 OR #2 OR #3 OR #4 OR #5 OR #6 OR #7 /(4619)
9. MeSH descriptor: [Benzodiazepines] explode all trees /(8955)
10. MeSH descriptor: [Antidepressive Agents, Tricyclic] explode all trees /(962)
11. MeSH descriptor: [Anticonvulsants] explode all trees /(2317)
12. MeSH descriptor: [Adrenergic alpha-Antagonists] explode all trees /(1220)
13. MeSH descriptor: [Antipsychotic Agents] explode all trees /(4387)
14. MeSH descriptor: [Antidepressive Agents] explode all trees /(5468)
15. (citalopram OR escitalopram OR fluoxetine OR fluvoxamine OR paroxetine OR sertraline OR desvenlafaxine OR venlafaxine OR duloxetine OR imipramine OR amitriptyline OR desipramine OR bupropion OR mirtazapine OR nefazodone OR trazodone OR prazosin OR olanzapine OR risperidone OR benzodiazepines OR alprazolam OR diazepam OR lorazepam OR clonazepam OR topiramate OR tiagabine OR lamotrigine OR carbamazepine OR divalproex):ti,ab,kw /(36277)
16. #9 OR #10 OR #11 OR #12 OR #13 OR #14 OR #15 /(46251)
17. #8 AND #16 /(498)

**4. PsycINFO <1806 to June 21, 2019>**

1. MA Stress Disorders, Post-Traumatic /(16987)
2. MA post-traumatic stress disorder /(16987)
3. MA post-traumatic stress disorders /(16987)
4. TI ((disorder* AND post-traumatic)) OR AB ((disorder* AND post-traumatic)) /(10056)
5. MA Stress Disorders, Traumatic /(17357)
6. MA Combat Disorders /(1886)
7. TI PTSD OR AB PTSD /(29688)
8. S1 OR S2 OR S3 OR S4 OR S5 OR S6 OR S7 /(39768)
9. MA Benzodiazepines /(5989)
10. MA Antidepressive Agents, Tricyclic /(3213)
11. MA Anticonvulsants /(7533)
12. MA Adrenergic alpha-Antagonists /(862)
13. MA Antipsychotic Agents /(23302)
14. MA Antidepressive Agents /(19646)
15. TI ((citalopram OR escitalopram OR fluoxetine OR fluvoxamine OR paroxetine OR sertraline OR desvenlafaxine OR venlafaxine OR duloxetine OR imipramine OR amitriptyline OR desipramine OR bupropion OR mirtazapine OR nefazodone OR trazodone OR prazosin OR olanzapine OR risperidone OR benzodiazepines OR alprazolam OR diazepam OR lorazepam OR clonazepam OR topiramate OR tiagabine OR lamotrigine OR carbamazepine OR divalproex)) OR AB ((citalopram OR escitalopram OR fluoxetine OR fluvoxamine OR paroxetine OR sertraline OR desvenlafaxine OR venlafaxine OR duloxetine OR imipramine OR amitriptyline OR desipramine OR bupropion OR mirtazapine OR nefazodone OR trazodone OR prazosin OR olanzapine OR risperidone OR benzodiazepines OR alprazolam OR diazepam OR lorazepam OR clonazepam OR topiramate OR tiagabine OR lamotrigine OR carbamazepine OR divalproex)) /(54806)
16. #9 OR #10 OR #11 OR #12 OR #13 OR #14 OR #15 /(697368)
17. #8 AND #16 /(5170)

**5. Ovid Health and Psychosocial Instruments <1985 to April 2019>**

1. (Stress Disorders, Post-Traumatic).mp. /(2500)
2. (post-traumatic stress disorder).mp. /(522)
3. (post-traumatic stress disorders).mp. /(13)
4. (disorder* AND post-traumatic).ti,ab. /(37)
5. (Stress Disorders, Traumatic).mp. /(8)
6. (Combat Disorders).mp. /(225)
7. PTSD.mp. /(1763)
8. #1 OR #2 OR #3 OR #4 OR #5 OR #6 OR #7 /(3650)
9. Benzodiazepines.mp. /(22)
10. (Antidepressive Agents, Tricyclic).mp. /(0)
11. Anticonvulsants.mp. /(8)
12. (Adrenergic alpha-Antagonists).mp. /(0)
13. (Antipsychotic Agents).mp. /(21)
14. (Antidepressive Agents).mp. /(19)
15. (citalopram OR escitalopram OR fluoxetine OR fluvoxamine OR paroxetine OR sertraline OR desvenlafaxine OR venlafaxine OR duloxetine OR imipramine OR amitriptyline OR desipramine OR bupropion OR mirtazapine OR nefazodone OR trazodone OR prazosin OR olanzapine OR risperidone OR benzodiazepines OR alprazolam OR diazepam OR lorazepam OR clonazepam OR topiramate OR tiagabine OR lamotrigine OR carbamazepine OR divalproex).mp. /(1616)
16. #9 OR #10 OR #11 OR #12 OR #13 OR #14 OR #15 /(1657)
17. #8 AND #16 /(34)

**6. Web of Science (ISI) <1970 to July 21, 2019>**

1. Topic: (PTSD) /(30212)

Databases= SCI-EXPANDED, SSCI, A&HCI, CPCI-S, CPCI-SSH, BKCI-S, BKCI-SSH, ESCI, CCR-EXPANDED, IC Timespan= 1970-2019

1. Topic: (posttraumatic) /(61003)

Databases= SCI-EXPANDED, SSCI, A&HCI, CPCI-S, CPCI-SSH, BKCI-S, BKCI-SSH, ESCI, CCR-EXPANDED, IC Timespan= 1970-2019

1. Topic: (post trauma) /(40167)

Databases= SCI-EXPANDED, SSCI, A&HCI, CPCI-S, CPCI-SSH, BKCI-S, BKCI-SSH, ESCI, CCR-EXPANDED, IC Timespan= 1970-2019

1. #3 OR #2 OR #1 /(94133)

Databases= SCI-EXPANDED, SSCI, A&HCI, CPCI-S, CPCI-SSH, BKCI-S, BKCI-SSH, ESCI, CCR-EXPANDED, IC Timespan= 1970-2019

1. Topic: (pharmacotherapy) /(45687)

Databases= SCI-EXPANDED, SSCI, A&HCI, CPCI-S, CPCI-SSH, BKCI-S, BKCI-SSH, ESCI, CCR-EXPANDED, IC Timespan= 1970-2019

1. #4 AND #5 /(875)

Databases= SCI-EXPANDED, SSCI, A&HCI, CPCI-S, CPCI-SSH, BKCI-S, BKCI-SSH, ESCI, CCR-EXPANDED, IC Timespan= 1970-2019

**Supplemental Table 1** The classification of drugs, abbreviation and pharmacological agents used in treating post-traumatic stress disorder

| **Classification of drugs** | **Abbreviations** | **Pharmacological agents** |
| --- | --- | --- |
| α1 receptor antagonist | / | Prazosin |
| Anticonvulsants | / | Divalproex, Lamotrigine, Pregabalin, Tiagabine, Topiramate |
| Antihistamine | / | Hydroxyzine |
| Atypical antipsychotics | / | Olanzapine, Quetiapine, Risperidone, Ziprasidone, Nafazodone |
| Beta blocker | / | Propranolol |
| Corticosteroid | / | Dexamethasone |
| CRF1 receptor antagonist | / | GSK561679 |
| Monoamine oxidase inhibitors | MAOIs | Brofaromine, Phenelzine |
| N-acetylcysteine | / | N-Acetylcysteine |
| Norepinephrine and dopamine reuptake inhibitors | NDRIs | Bupropion |
| Neurokinin-1 receptor antagonist | NK1 | GR205171 |
| Norepinephrine reuptake inhibitor | NRI | Reboxetine |
| Reversible cholinesterase inhibitor | / | Rivastigmine |
| Serotonin modulator and stimulators | SMSs | Vilazodone |
| Serotonin and norepinephrine reuptake inhibitors | SNRIs | Venlafaxine |
| Selective serotonin reuptake inhibitors | SSRIs | Citalopram, Fluoxetine, Paroxetine, Sertraline, Fluvoxamine |
| Tricyclic antidepressants | TCAs | Amitriptyline, Imipramine |
| Tetracyclic antidepressants | TeCAs | Mirtazapine |
| α2A receptor agonist | / | Guanfacine |

**Supplemental Table 2** Funding source of included studies

| **Study** | **Funding** |
| --- | --- |
| Ahmadpanah 2014 | Not reported |
| Akuchekian 2004 | Not reported |
| Back 2016 | Department of Defense grant. |
| Baker 1995 | Not reported |
| Baniasadi 2014 | Not reported |
| Bartzokis2005 | Janssen Research Foundation (GB),the Department of Veterans Affairs. |
| Batki 2014 | The Department of Defense W81XWH-05-2-0094, W81XWH-12-2-0137, and W81XWH-11-2-0245 (SLB). |
| Becker 2007 | GlaxoSmithKline and Veterans Affairs Merit Awards MH-0018,R01CA81595, R01MH062482, and K24DA016388. |
| Brady 2000 | Pfizer. |
| Brunet A 2018 | Not reported |
| Butterfield 2001 | Eli Lilly. |
| Carey 2012 | Eli Lilly. |
| Connor 1999 | NIMH IROI-MH44740-01 Eli Lilly. |
| Davidson 1990 | Veterans Administration grant. |
| Davidson 2001 | Pfizer. |
| Davidson 2003 | Organon. |
| Davidson 2006a | Wyeth Pharmaceuticals. |
| Davidson 2006b | Wyeth Pharmaceuticals Pfizer, Solvay, Eli Lilly, GlaxoSmithKline, Wyeth, Organon, Forest, PureWorld, Allergan, Nutrition 21, Bristol Myers Squibb, Johnson and Johnson, Cephalon, AstraZeneca, Parke Davis, Pharmacia, Upjohn. |
| Davidson 2007 | Cephalon. |
| Davis 2004 | Bristol-Myers Squibb Pfizer. |
| Davis 2008a | VA Abbott Laboratories. |
| Davis 2008b | VA Merit Award. |
| Dunlop 2017 | National Institute of Mental Health, U19 MH069056 (BWD, HM) K23 MH086690 (BWD) and VA CSRD GlaxoSmithKline lilly. |
| Friedman 2007 | Pfizer Inc. |
| Hamner 2003 | Janssen Pharmaceu tica. |
| Hamner 2009 | Abbott Laborato ries, AstraZeneca, and Otsuka Phar maceutical. |
| Hertzberg 1999 | Glaxo Wellcome Inc. and a R29 MH51752-01 awarded by NIMH to JCB. |
| Hertzberg 2000 | Eli Lilly, NIMH Grant. |
| Hodgins 2018 | Icahn School of Medicine at Mount Sinai from Alkermes National Institutes of Health, Department of Veterans Affairs, Johnson Family Chair the National Institute of Mental Health, Department of Defense, and Department of Veterans Affairs and Assurex Acadia P.D.H. has received research support from Takeda H.S.M. has received consulting fees from St Jude Medical Neuromodulation and Eli Lilly (2013 only) and intellectual property licensing fees from St Jude Medical Neuromodulation. |
| Katz 1994 | Ciba-Geigy Corporation. |
| Kosten 1991 | Supported in part by the Veterans Administration Research Funds and Research Scientist Award DA00112 to Dr. Kosten. |
| Krystal 2011 | VA Cooperative Cooperative Studies Program of the Department of Veterans Affairs Office Ortho-McNeil Janssen Scientific Affairs. |
| Li ‎2017 | The Science and Technology Talents Program of Harbin (2014RFXGJ041, 2014RFQGJ094); Post Doctoral Fund (160780); Harbin High Level Talent Fund (HRBGCCRCJJ-6, 2013SYYRCYJ01-1); Heilongjiang Natural Science Foundation (QC 2016102, H 2016002). |
| Lindley 2007 | Ortho-McNeil Pharmaceutical, Inc. |
| Mahabir 2016 | Canadian Institutes of Health Research a post-doctoral fellow-ship from Fonds de recherché en santé Quebec. |
| Marshall 2001 | GlaxoSmithKline and NIMH grant MH-01412. |
| Marshall 2007 | National Institutes of Mental Health Glaxo SmithKline. |
| Martenyi 2002 | Eli Lilly. |
| martenyi 2007 | Eli Lilly. |
| Mathew 2001 | NIMH Grant U19 MH69056 and the Intramural Research Program at the National Institute of Mental Health (NIMH). |
| McRae 2004 | Bristol-Myers Squibb. |
| Monnelly 2003 | Janssen Foundation Veterans Affairs Medical Research Service. |
| Naylor 2015 | VA Mid-Atlantic MIRECC, Department of Veterans Affairs Advanced Research Career Development Award (Marx), and VA Career Development Transition Award (Marx). Dr Naylor is supported by a Department of Veterans Affairs Rehabilitation Research and Development Career Development Award (1lK2RX000908). Bristol Meyers Squibb generously donated aripiprazole. |
| Neylan 2006 | Pfizer and Forest Pharmaceuticals. VA Sierra Pacific. |
| Padala 2006 | Janssen Pharmaceuticals. |
| Panahi 2011 | Baqiyatallah University of Medical Sciences. |
| Petrakis 2016 | Department of Defense (W81XWH-08-2-0075-PT075375). |
| Ramaswamy 2016 | Pfizer. |
| Ramaswamy ‎2017 | Forest Laboratories. |
| Raskind 2007 | Department of Veterans Affairs and NIH R01MH069867. |
| Raskind 2013 | The Department of Veterans Affairs, U.S. Army Medical Research and Materiel Command, Fort Detrick, Md.; and by NIH grant 1R01MH069867 Lilly Pharmaceuticals. |
| Raskind 2018 | The Department of Veterans Affairs Cooperative Studies Program. Pfizer. |
| Reich 2004 | Janssen Pharmaceutica. |
| Rezaei 2017 | Not reported |
| Rothbaum 2008 | Janssen and Wyeth. |
| Spivak 2006 | Medical Corps of the Israel Defense Force and the Agis Pharmaceutics Company (Ramat Gan, Israel). |
| Stein 2002 | Lily. |
| Surís 2017 | Not reported |
| Tucker 2001 | SmithKline Beecham Pharmaceuticals Pfizer lilly. |
| Tucker 2003 | Forest Pharmaceuticals Inc. |
| Tucker 2007 | Ortho-McNeil AstraZeneca, Bristol-Myers Squibb, Ortho-McNeil, Otsuka, Pfizer, GlaxoSmithKline, and Cephalon. |
| van der Kolk ‎1994 | Eli Lilly and company indianapolis. |
| van der Kolk 2007 | R01MH58363 from the National Institute of Mental Health. |
| Villarreal 2016 | Alkermes and Pfizer. |
| Yeh ‎2011 | Fundac¸ao de Amparo a Pesquisa do Estado`de Sao Paulo (grant: 2004/15039-0); MCPC received a scholarship˜from the Ministry of Education (CAPES grant: 27909024886). |
| Zohar ‎2002 | Pfizer. |

**Supplemental Table 3** Risk of bais for included studies

| **Study, Year** | **Random sequence generation**  **(Selection bias)** | **Allocation concealment**  **(Selection bias)** | **Blinding of participants and personnel**  **(Performance bias)** | **Blinding of outcome assessment**  **(Detection bias)** | **Selective reporting (Reporting bias)** | **Incomplete outcome data (Attrition bias)** | **Other bias** |
| --- | --- | --- | --- | --- | --- | --- | --- |
| Ahmadpanah 2014 | Low risk | Low risk | Low risk | Low risk | Unclear | High risk | Unclear |
| Akuchekian 2004 | Unclear | Low risk | Low risk | Unclear | Unclear | Low risk | Unclear |
| Back 2016 | Unclear | Low risk | Low risk | Unclear | Unclear | Low risk | Unclear |
| Baker 1995 | Unclear | Unclear | Low risk | Unclear | Unclear | High risk | Unclear |
| Baniasadi 2014 | Unclear | Unclear | Low risk | Unclear | Unclear | Low risk | Unclear |
| Batki 2014 | Low risk | Low risk | Low risk | Low risk | Low risk | Low risk | Unclear |
| Becker 2007 | Unclear | Unclear | Low risk | Unclear | Unclear | Low risk | Unclear |
| Brady 2000 | Unclear | Unclear | Low risk | Unclear | Unclear | Low risk | Unclear |
| Brunet 2018 | Low risk | Unclear | Low risk | Unclear | Unclear | Low risk | Unclear |
| Butterfield 2001 | Unclear | Unclear | Low risk | Unclear | Unclear | Low risk | Unclear |
| Carey 2012 | Low risk | Low risk | Low risk | Unclear | Low risk | Low risk | Unclear |
| Connor 1999 | Low risk | Low risk | Low risk | Low risk | Unclear | Low risk | Unclear |
| Davidson 1990 | Unclear | Unclear | Low risk | Unclear | Unclear | Low risk | Unclear |
| Davidson 2001 | Low risk | Unclear | Low risk | Unclear | Unclear | Low risk | Unclear |
| Davidson 2003 | Unclear | Unclear | Low risk | Unclear | Unclear | Low risk | Unclear |
| Davidson 2006a | Unclear | Unclear | Low risk | Unclear | Unclear | Low risk | Unclear |
| Davidson 2006b | Low risk | Unclear | Low risk | Unclear | Unclear | Low risk | Unclear |
| Davidson 2007 | Unclear | Unclear | Low risk | Unclear | Unclear | Low risk | Unclear |
| Davis 2004 | Unclear | Low risk | Low risk | Unclear | Unclear | Low risk | Unclear |
| Davis 2008 | Low risk | Low risk | Low risk | Low risk | Unclear | Low risk | Unclear |
| Dunlop 2017 | Low risk | Low risk | Low risk | Unclear | Unclear | Low risk | Unclear |
| Friedman 2007 | Low risk | Unclear | Low risk | Unclear | Unclear | Low risk | Unclear |
| Hamner 2003 | Unclear | Unclear | Low risk | Low risk | Unclear | Low risk | Unclear |
| Hamner 2009 | Unclear | Unclear | Low risk | Low risk | Unclear | Low risk | Unclear |
| Hertzberg 1999 | Unclear | Unclear | Low risk | Unclear | Unclear | Low risk | Unclear |
| Hertzberg 2000 | Unclear | Unclear | Low risk | Unclear | Unclear | Low risk | Unclear |
| Hodgins 2018 | Unclear | Unclear | Low risk | Low risk | Unclear | High risk | Unclear |
| Katz 1995 | Unclear | Unclear | Low risk | Unclear | Unclear | Low risk | Unclear |
| Kosten 1991 | Unclear | Unclear | Low risk | Low risk | Unclear | Low risk | Unclear |
| Krystal 2011 | Low risk | Unclear | Low risk | Unclear | Unclear | Low risk | Unclear |
| Lindley 2007 | Unclear | Unclear | Low risk | Low risk | Unclear | Low risk | Unclear |
| Mahabir 2016 | Unclear | Unclear | Low risk | Unclear | Unclear | Low risk | Unclear |
| Marshall 2001 | Unclear | Unclear | Low risk | Unclear | Unclear | Low risk | Unclear |
| Marshall 2007 | Unclear | Unclear | Low risk | Low risk | Unclear | Low risk | Unclear |
| Martenyi 2002 | Low risk | Low risk | Low risk | Low risk | Unclear | Low risk | Unclear |
| Martenyi 2007 | Unclear | Unclear | Low risk | High risk | Unclear | Low risk | Unclear |
| McRae 2004 | Unclear | Unclear | Low risk | Unclear | Unclear | Low risk | Unclear |
| Monnelly 2003 | Unclear | Unclear | Low risk | Low risk | Unclear | Low risk | Unclear |
| Yeh ‎2011 | Low risk | Low risk | Low risk | Low risk | Unclear | Low risk | Unclear |
| Naylor 2015 | Unclear | Unclear | Low risk | Unclear | Low risk | Low risk | Unclear |
| Neylan 2006 | Unclear | Unclear | Low risk | Low risk | Unclear | Low risk | Unclear |
| Padala 2006 | Unclear | Unclear | Low risk | Unclear | Unclear | Low risk | Unclear |
| Panahi 2011 | Low risk | Unclear | Low risk | Low risk | Unclear | Low risk | Unclear |
| Petrakis 2016 | Unclear | Unclear | Low risk | Unclear | Low risk | Low risk | Unclear |
| Ramaswamy 2016 | Unclear | Unclear | Low risk | Low risk | Unclear | Low risk | Unclear |
| Ramaswamy ‎2017 | Low risk | Unclear | Low risk | Low risk | Unclear | Low risk | Unclear |
| Raskind 2007 | Low risk | Unclear | Low risk | Low risk | Unclear | Low risk | Unclear |
| Raskind 2013 | Unclear | Low risk | Low risk | Low risk | High risk | Low risk | Unclear |
| Raskind 2018 | Low risk | Low risk | Low risk | Low risk | Low risk | Low risk | Unclear |
| Reich 2004 | Unclear | Unclear | Low risk | Unclear | Unclear | Low risk | Unclear |
| Rezaei 2017 | Low risk | Low risk | Low risk | Low risk | Unclear | Low risk | Unclear |
| Rothbaum 2008 | Unclear | Unclear | Low risk | Unclear | Unclear | Low risk | Unclear |
| Spivak 2006 | Low risk | Low risk | Low risk | Low risk | Unclear | Low risk | Unclear |
| Suris 2017 | Unclear | Unclear | Low risk | Unclear | Unclear | Low risk | Unclear |
| Tucker 2001 | Unclear | Unclear | Low risk | Unclear | Unclear | Low risk | Unclear |
| Tucker 2003 | Unclear | Unclear | Low risk | Low risk | Unclear | Low risk | Unclear |
| Tucker 2007 | Low risk | Low risk | Low risk | Low risk | Unclear | Low risk | Unclear |
| Van Der Kolk ‎1994 | Unclear | Unclear | Low risk | Unclear | Unclear | Low risk | Unclear |
| Van Der Kolk 2007 | Unclear | Unclear | Low risk | Low risk | Unclear | Low risk | Unclear |
| Villarreal 2016 | Low risk | Unclear | Low risk | Unclear | Low risk | Low risk | Unclear |
| Li ‎2017 | Low risk | Low risk | Low risk | Low risk | Low risk | Low risk | Unclear |
| Zohar ‎2002 | Unclear | Unclear | Low risk | Low risk | Unclear | Low risk | Unclear |
| Bartzokis2005 | Unclear | Unclear | Low risk | Low risk | Unclear | Low risk | Unclear |
| Mathew 2011 | Unclear | Low risk | Low risk | Low risk | Unclear | High risk | Unclear |
| Stein 2002 | Unclear | Unclear | Low risk | Unclear | Unclear | Unclear | Unclear |
| Davis 2008b | Unclear | Unclear | Low risk | Unclear | Unclear | Low risk | Unclear |

**Supplemental Table 4** Placebo-controlled comparisons for efficacy and acceptability of pharmacological management for PTSD

| **Active drugs** | | **PTSD total symptoms (clinician-rated)** | **Re-experiencing** | **Avoidance** | **Hyperarousal** | **Depression** | **Anxiety** | **All-cause discontinuation** | **Discontinuation due to adverse effect** |
| --- | --- | --- | --- | --- | --- | --- | --- | --- | --- |
| All active drugs | | **N=57, n1=3124,n2=2811, SMD=-0.33,95%CI:-0.43,-0.23,I^2^=65%** | **N=36, n1=2362,n2=2065, SMD=-0.32,95%CI:-0.41,-0.23,I^2^=43%** | **N=40, n1=2432,n2=2121, SMD=-0.27,95%CI:-0.33,-0.21,I^2^=23%** | **N=40, n1=2432,n2=2121, SMD=-0.28,95%CI:-0.34,-0.22,I^2^=30%** | **N=44, n1=2635,n2=2323, SMD=-0.28,95%CI:-0.34,-0.23,I^2^=29%** | **N=19, n1=1108,n2=815, SMD=-0.23,95%CI:-0.33,-0.14,I^2^=35%** | N=61, n1=3137,n2=2930, RR=0.95,95%CI:0.88,1.03,I^2^=4% | **N=62, n1=3249,n2=2916, RR=1.47,95%CI:1.24,1.75,I^2^=0%** |
| Alpha blockers | Prazosin | N=5, n1=264,n2=265, SMD=-0.59,95%CI:-1.23,0.04,I^2^=90% | N=2, n1=82,n2=81, SMD=-0.52,95%CI:-1.66,0.63,I^2^=92% | N=2, n1=82,n2=81, SMD=-0.32,95%CI:-1.07,0.43,I^2^=82% | N=2, n1=82,n2=81, SMD=-0.21,95%CI:-0.82,0.40,I^2^=73% | **N=2 n1=46,n2=50, SMD=-0.54,95%CI:-0.95,-0.13,I^2^=0%** | NA | N=4, n1=254,n2=253, RR=0.82,95%CI:0.56,1.22,I^2^=2% | N=4, n1=135,n2=134, RR=1.00,95%CI:0.08,12.83,I^2^=49% |
| Anticonvulsants | Overall | N=7, n1=240,n2=232, SMD=-0.31,95%CI:-0.79,0.17,I^2^=82% | N=4, n1=87,n2=84, SMD=-0.02,95%CI:-0.32,0.28,I^2^=0% | N=5, n1=98,n2=88, SMD=0.03,95%CI:-0.27,0.33,I^2^=9% | N=5, n1=98,n2=88, SMD=-0.01,95%CI:-0.56,0.53,I^2^=64% | N=6, n1=119,n2=121, SMD=-0.13,95%CI:-0.38,0.13,I^2^=0% | N=4, n1=93,n2=92, SMD=-0.07,95%CI:-0.36,0.22,I^2^=13% | N=10, n1=304,n2=295, RR=0.87,95%CI:0.67,1.11,I^2^=19% | N=10, n1=304,n2=295, RR=1.54,95%CI:0.91,2.60,I^2^=0% |
|  | Tiagabine | N=1, n1=105,n2=97, SMD=0.02,-0.26,0.30 | NA | NA | NA | NA | NA | N=1, n1=116,n2=116, RR=0.75,95%CI:0.54,1.04 | N=1 n1=116,n2=116, RR=1.00,95%CI:0.41,2.43 |
|  | Lamotrigine | NA | NA | N=1 n1=11,n2=4 | N=1 n1=11,n2=4 | NA | NA | N=1, n1=10,n2=4, RR=0.27,95%CI:0.07,1.04 | N=1, n1=10,n2=4, RR=0.40,95%CI:0.08,1.94 |
|  | Divalproex | N=2, n1=56,n2=54, SMD=0.19,-0.38,0.75,I2=45% | N=2, n1=56,n2=54, SMD=0.03,95%CI:-0.34,0.41,I^2^=0% | N=2, n1=56,n2=54, SMD=0.23,95%CI:-0.14,0.61,I^2^=0% | N=2, n1=56,n2=54, SMD=0.35,95%CI:-0.80,1.50,I^2^=85% | N=2,, n1=56,n2=54, SMD=-0.19,95%CI:-0.57,0.18,I^2^=0% | N=2, n1=56,n2=54, SMD=0.16,95%CI:-0.43,0.74,I^2^=48% | N=2, n1=56,n2=54, RR=0.87,95%CI:0.46,1.62,I^2^=0% | N=2, n1=56,n2=54, RR=2.31,95%CI:0.48,11.23,I^2^=0% |
|  | Pregabalin | NA | NA | NA | NA | N=1, n1=18,n2=19,SMD=0.22,95%CI:-0.43,0.86 | N=1, n1=18,n2=19,SMD=-0.26,95%CI:-0.90,0.39 | N=1, n1=18,n2=19 | N=1, n1=18,n2=19 |
|  | Topiramate | N=4, n1=79,n2=81, SMD=-0.71,-1.42,0.00,I^2^=77% | N=2, n1=31,n2=30, SMD=-0.13,95%CI:-0.63,0.57,I^2^=47% | N=2, n1=31,n2=30, SMD=-0.34,95%CI:-0.84,0.17,I^2^=0% | N=2, n1=31,n2=30, SMD=-0.36,95%CI:-0.86,0.15,I^2^=0% | N=3, n1=45,n2=48, SMD=-0.19,95%CI:-0.60,0.23,I^2^=0% | N=1, n1=19,n2=19, SMD=-0.31,95%CI:-0.95,0.33 | N=5, n1=104,n2=102, RR=1.44,95%CI:0.81,2.56,I^2^=0% | **N=5, n1=104,n2=102, RR=2.61,95%CI:1.09,6.25,I^2^=0%** |
| Atypical antipsychotics | Overall | **N=12, n1=304,n2=294, SMD=-0.30,95%CI:-0.46,-0.13,I^2^=0%** | **N=8, n1=267,n2=258, SMD=-0.37,95%CI:-0.54,-0.20,I^2^=21%** | **N=8, n1=267,n2=258, SMD=-0.37,95%CI:-0.54,-0.19,I^2^=0%** | N=8, n1=267,n2=258, SMD=-0.11,95%CI:-0.38,0.15,I2=40% | **N=7, n1=246,n2=244, SMD=-0.33,95%CI:-0.51,-0.15,I^2^=15%** | **N=4, n1=208,n2=210, SMD=-0.32,95%CI:-0.51,-0.12,I^2^=11%** | N=12 n1=313,n2=295, RR=1.07,95%CI:0.79,1.46,I^2^=9% | **N=13, n1=327,n2=309, RR=2.06,95%CI:1.10,3.84,I^2^=0%** |
|  | Quetiapine | **N=1, n1=42,n2=38, SMD=-0.49,95%CI:-0.93, -0.04** | **N=1, n1=42,n2=38, SMD=-0.55,95%CI:-1.00,-0.10** | **N=1, n1=42,n2=38, SMD=-0.53,95%CI:-0.98, -0.09** | N=1, n1=42,n2=38, SMD=-0.24,95%CI:-0.68,0.20 | **N=1, n1=42,n2=38, SMD=-0.63,95%CI:-1.08,-0.18** | N=1, n1=42,n2=38, SMD=-0.41,95%CI:-0.86,0.03 | N=1, n1=42,n2=38, RR=0.59,95%CI:0.34,1.01 | N=1, n1=42,n2=38, RR=2.71,95%CI:0.79,9.29 |
|  | Aripiprazole | N=1,n1=7,n2=7, SMD=-0.33,95%CI:-1.39, 0.72 | NA | NA | NA | NA | NA | N=1, n1=7,n2=7, RR=5,95%CI:0.29,87.54 | N=1, n1=7,n2=7 |
|  | Risperidone | **N=6, n1=206,n2=206, SMD=-0.23,95%CI:-0.42, -0.03,I^2^=0%** | N=4, n1=186,n2=186, SMD=-0.36,95%CI:-0.74,0.01,I^2^=49% | **N=4, n1=186,n2=186, SMD=-0.36,95%CI:-0.56, -0.15,I^2^=0%** | N=4, n1=186,n2=186, SMD=0.09,95%CI:-0.11,0.30,I^2^=0% | N=3, n1=165,n2=168, SMD=-0.19,95%CI:-0.41,0.03,I^2^=0% | N=2, n1=151,n2=157, SMD=-0.44,95%CI:-1.02,0.14,I^2^=67% | N=7, n1=229,n2=221, RR=1.37,95%CI:0.89,2.11,I^2^=0% | N=7, n1=229,n2=221, RR=2.26,95%CI:0.78,6.61,I^2^=0% |
|  | Olanzapine | **N=3, n1=34,n2=28, SMD=-0.66,95%CI:-1.19,-0.13,I^2^=34%** | N=2, n1=24,n2=19, SMD=-0.25,95%CI:-1.16,0.66,I^2^=49% | N=2, n1=24,n2=19, SMD=-0.34,95%CI:-1.29,0.61,I^2^=53% | N=2, n1=24,n2=19, SMD=-0.62,95%CI:-1.78,0.53,I^2^=66% | **N=2, n1=24,n2=23, SMD=-0.81,95%CI:-1.41,-0.20,I^2^=0%** | NA | N=2, n1=20,n2=14, RR=1.41,95%CI:0.41,4.78,I^2^=0% | N=3, n1=34,n2=28, RR=3.38,95%CI:0.62,18.51,I^2^=0% |
|  | Ziprasidone | N=1, n1=15,n2=15, SMD=-0.04,95%CI:-0.76,0.67 | N=1, n1=15,n2=15, SMD=-0.13,95%CI:-0.84,0.59 | N=1, n1=15,n2=15, SMD=0.03,95%CI:-0.69,0.74 | N=1, n1=15,n2=15, SMD=-0.02,95%CI:-0.74,0.70 | N=1, n1=15,n2=15, SMD=-0.49,95%CI:-1.22,0.24 | N=1, n1=15,n2=15, SMD=-0.43,95%CI:-1.16,0.29 | N=1, n1=15,n2=15, RR=0.75,95%CI:0.20,2.79 | N=1, n1=15,n2=15, RR=0.75,95%CI:0.20,2.79 |
| Beta blocker | Propranolol | N=1, n1=30,n2=30, SMD=-0.25,95%CI:-0.75, 0.26 | NA | NA | NA | NA | NA | N=1, n1=30,n2=30, RR=1.00,95%CI:0.60,1.66 | N=1, n1=30,n2=30 |
| Corticosteroid | Dexamethasone | NA | NA | NA | NA | N=1, n1=26,n2=28, SMD=-0.14,95%CI:-0.67,0.40 | NA | N=1, n1=26,n2=28, RR=1.97,95%CI:0.85,4.57 | N=1, n1=26,n2=28, RR=3.23,95%CI:0.14,75.79 |
| CRF1 receptor antagonist | GSK561679 | N=2, n1=126,n2=130, SMD=0.09,95%CI:-0.15, 0.34,I^2^=0% | NA | NA | NA | N=1, n1=63,n2=65, SMD=-0.19,95%CI:-0.54,0.16 | NA | N=1, n1=63,n2=65, RR=1.03,95%CI:0.57,1.88 | N=1, n1=63,n2=65, RR=2.75,95%CI:0.76,9.90 |
| MAOI | Overall | N=1, n1=33,n2=31, SMD=-0.28,95%CI:-0.07, 0.22 | NA | **N=1, n1=19,n2=18, SMD=-1.07,95%CI:-1.77, -0.38** | **N=1, n1=19,n2=18, SMD=-0.81,95%CI:-1.49, -0.14** | N=1, n1=19,n2=18, SMD=-0.40,95%CI:-1.06, 0.25 | **N=1, n1=19,n2=18, SMD=-0.67,95%CI:-1.34, -0.01** | N=2, n1=52,n2=49, RR=0.66,95%CI:0.16,2.61,I^2^=81% | N=3, n1=108,n2=107, RR=1.33,95%CI:0.60,2.93,I^2^=9% |
|  | Brofaromine | N=1, n1=33,n2=31, SMD=-0.28,95%CI:-0.07, 0.22 | NA | NA | NA | NA | NA | N=1 n1=33,n2=31, RR=1.29,95%CI:0.60,2.78 | N=2, n1=89,n2=89, RR=1.81,95%CI:0.74,4.48,I^2^=0% |
|  | Phenelzine | NA | NA | **N=1, n1=19,n2=18, SMD=-1.07,95%CI:-1.77,-0.38** | **N=1, n1=19,n2=18, SMD=-0.81,95%CI:-1.49,-0.14** | N=1, n1=19,n2=18, SMD=-0.40,95%CI:-1.06,0.25 | **N=1 n1=19,n2=18, SMD=-0.67,95%CI:-1.34,-0.01** | **N=1, n1=19,n2=18, RR=0.32,95%CI:0.12,0.80** | N=1, n1=19,n2=18,RR=0.32,95%CI:0.04,2.76 |
| N-acetylcysteine | Overall | N=1, n1=13,n2=14, SMD=-0.54,95%CI:-1.31, 0.23 | N=1, n1=13,n2=14, SMD=-0.15,95%CI:-0.91, 0.60 | N=1, n1=13,n2=14, SMD=-0.20,95%CI:-0.96, 0.55 | N=1, n1=13,n2=14, SMD=-0.61,95%CI:-1.38, 0.17 | N=1, n1=13,n2=14, SMD=-0.74,95%CI:-1.53, 0.04 | NA | N=1, n1=18,n2=17, RR=1.57,95%CI:0.44,5.60 | N=1 n1=18,n2=17 |
| NDRIs | Bupropion | N=1, n1=15,n2=7, SMD=0.21,95%CI:-0.69,1.11 | N=1, n1=18,n2=10, SMD=-0.18,95%CI:-0.59, 0.96 | N=1, n1=18,n2=10, SMD=-0.34,95%CI:-1.12, 0.43 | N=1, n1=18,n2=10, SMD=-0.10,95%CI:-0.88, 0.67 | N=1, n1=18,n2=10, SMD=0.05,95%CI:-0.72, 0.83 | NA | NA | N=1, n1=18,n2=10, RR=1.70,95%CI:0.08,38.17 |
| NK1 receptor antagonist | GR205171 | N=1, n1=20,n2=19, SMD=-0.24,95%CI:-0.87,0.39 | NA | NA | NA | NA | NA | N=1, n1=20,n2=19, RR=0.32,95%CI:0.07,1.38 | N=1, n1=20,n2=19 |
| Reversible cholinesterase inhibitor | Rivastigmine | NA | N=1, n1=12,n2=12, SMD=-0.33,95%CI:-1.13, 0.48 | N=1, n1=12,n2=12, SMD=-0.18,95%CI:-0.98, 0.62 | N=1, n1=12,n2=12, SMD=-0.41,95%CI:-1.22, 0.40 | NA | NA | NA | N=1, n1=12,n2=12 |
| Antihistamine | Hydroxyzine | **N=1, n1=34,n2=34,SMD=-1.56, [-2.11,-1.02** | NA | NA | NA | NA | NA | NA | N=1, n1=34,n2=33 |
| SARIs | Nefazodone | N=1, n1=26,n2=15,SMD=-0.23, [-0.86,0.41 | N=1, n1=26,n2=15, SMD=-0.34,95%CI:-0.98, 0.30 | N=1, n1=26,n2=15,SMD=-0.15, [-0.79,0.48 | N=1, n1=26,n2=15, SMD=-0.13,95%CI:-0.77, 0.51 | N=1, n1=26,n2=15, SMD=-0.27,95%CI:-0.91, 0.37 | NA | N=1, n1=26,n2=15, RR=1.15,95%CI:0.55,2.43 | N=1, n1=26,n2=25, RR=2.88,95%CI:0.37,22.43 |
| SMSs | Vilazodone | NA | NA | NA | NA | N=1, n1=23,n2=24, SMD=0.06,95%CI:-0.51, 0.63 | N=1, n1=22,n2=24, SMD=-0.21,95%CI:-0.79, 0.37 | N=1, n1=29,n2=30, RR=0.52,95%CI:0.17,1.53 | N=1, n1=29,n2=30, RR=3.10,95%CI:0.13,73.13 |
| SNRIs | Venlafaxine | **N=2, n1=340,n2=347, SMD=-0.29,95%CI:-0.44,-0.14,I^2^=0%** | **N=2, n1=340,n2=347, SMD=-0.25,95%CI:-0.40, -0.10,I^2^=0%** | **N=2, n1=340,n2=347, SMD=-0.20,95%CI:-0.35, -0.05,I^2^=0%** | **N=2, n1=340,n2=347, SMD=-0.28,95%CI:-0.43, -0.13,I^2^=0%** | **N=2, n1=340,n2=347, SMD=-0.21,95%CI:-0.36, -0.06,I^2^=0%** | NA | N=2, n1=340,n2=347, RR=0.86,95%CI:0.69,1.07,I^2^=0% | N=2, n1=340,n2=347, RR=1.19,95%CI:0.62,2.26,I^2^=40% |
| SSRIs | Overall | **N=18 n1=1598,n2=1317, SMD=-0.33,95%CI:-0.40,-0.25,I^2^=39%** | **N=15, n1=1499,n2=1227, SMD=-0.37,95%CI:-0.49, -0.25,I^2^=53%** | **N=15, n1=1499,n2=1227, SMD=-0.28,95%CI:-0.38, -0.17,I^2^=36%** | **N=15, n1=1499,n2=1227, SMD=-0.34,95%CI:-0.41, -0.26,I^2^=0%** | **N=12, n1=1592,n2=1293, SMD=-0.28,95%CI:-0.41, -0.16,I^2^=57%** | N=6, n1=709,n2=428, SMD=-0.16,95%CI:-0.34, 0.01,I^2^=47% | N=19, n1=1550,n2=1388, RR=0.99,95%CI:0.89,1.10,I^2^=0% | **N=16, n1=1665,n2=1383, RR=1.37,95%CI:1.08,1.74,I^2^=0%** |
|  | Sertraline | **N=6,n1=494,n2=484, SMD=-0.22,95%CI:-0.35,-0.10,I^2^=39%** | **N=7,n1=481,n2=473, SMD=-0.45,95%CI:-0.75,-0.16,I^2^=76%** | **N=7,n1=481,n2=473, SMD=-0.26,95%CI:-0.47,-0.06,I^2^=51%** | **N=7,n1=481,n2=473, SMD=-0.33,95%CI:-0.46,-0.20,I^2^=0%** | N=6,n1=494,n2=484, SMD=-0.18,95%CI:-0.39,0.03,I^2^=55% | N=2,n1=182,n2=186, SMD=0.02,95%CI:-0.34,0.38,I^2^=68% | N=8, n1=565,n2=555, RR=1.07,95%CI:0.89,1.29,I^2^=0% | N=7, n1=542,n2=545, RR=1.47,95%CI:0.99,2.20,I^2^=0% |
|  | Fluoxetine | **N=6,n1=521,n2=260, SMD=-0.27,95%CI:-0.42,-0.12,I^2^=27%** | **N=3,n1=460,n2=203, SMD=-0.27,95%CI:-0.43,-0.10,I^2^=0%** | **N=3,n1=460,n2=203, SMD=-0.24,95%CI:-0.41,-0.08,I^2^=0%** | **N=3,n1=460,n2=203, SMD=-0.20,95%CI:-0.37,-0.04,I^2^=0%** | **N=4,n1=540,n2=258,SMD=-0.25,95%CI:-0.40,-0.10,I^2^=0%** | **N=3,n1=510,n2=229,SMD=-0.28,95%CI:-0.44,-0.12,I^2^=0%** | N=6, n1=419,n2=268, RR=0.90,95%CI:0.69,1.17,I^2^=36% | N=5, n1=582,n2=195, RR=1.05,95%CI:0.61,1.81,I^2^=20% |
|  | Paroxetine | **N=4,n1=533,n2=541, SMD=-0.48,95%CI:-0.60,-0.36,I^2^=0%** | **N=4,n1=533,n2=541, SMD=-0.40,95%CI:-0.52,-0.27,I^2^=0%** | **N=4,n1=533,n2=541, SMD=-0.39,95%CI:-0.51,-0.27,I^2^=0%** | **N=4,n1=533,n2=541, SMD=-0.42,95%CI:-0.54,-0.30,I^2^=0%** | **N=4,n1=533,n2=541, SMD=-0.49,95%CI:-0.61,-0.36,I^2^=7%** | N=1,n1=17,n2=13, SMD=-0.18,95%CI:-0.90,0.54 | N=4, n1=541,n2=555, RR=0.97,95%CI:0.83,1.13,I^2^=0% | **N=4, n1=541,n2=369, RR=1.45,95%CI:1.02,2.07,I^2^=0%** |
|  | Citalopram | N=1, n1=25,n2=10, SMD=0.17,95%CI:-0.56,0.91 | N=1, n1=25,n2=10, SMD=0.11,95%CI:-0.62,0.85 | N=1, n1=25,n2=10, SMD=0.16,95%CI:-0.58,0.89 | N=1, n1=25,n2=10, SMD=0.16,95%CI:-0.57,0.90 | N=1, n1=25,n2=10, SMD=-0.18,95%CI:-0.91,0.56 | NA | N=1, n1=25,n2=10, RR=0.67,95%CI:0.20,2.28 | NA |
|  | Valizodone | N=1, n1=25,n2=22, SMD=-0.12,95%CI:-0.69,0.45 | NA | NA | NA | NA | NA | NA | NA |
| TCAs | Overall | N=1, n1=17,n2=16, SMD=-0.63,95%CI:-1.33,0.07 | NA | N=2, n1=40,n2=34, SMD=-0.43,95%CI:-0.89, 0.04,I^2^=27% | N=2, n1=40,n2=34, SMD=-0.55,95%CI:-1.18,0.09,I^2^=44% | N=2, n1=40,n2=34, SMD=-0.67,95%CI:-1.58,0.25,I^2^=72% | **N=2, n1=40,n2=34, SMD=-0.68,95%CI:-1.16, -0.21,I^2^=16%** | N=2, n1=48,n2=39, RR=0.94,95%CI:0.59,1.50,I^2^=4% | N=2, n1=48,n2=39, RR=1.72,95%CI:0.53,5.61,I^2^=17% |
|  | Amitriptyline | N=1, n1=17,n2=16, SMD=-0.63,95%CI:-1.33,0.07 | NA | **N=1, n1=17,n2=16, SMD=-0.75,95%CI:-1.46,-0.04** | **N=1, n1=17,n2=16, SMD=-0.90,95%CI:-1.62,-0.18** | **N=1, n1=17,n2=16, SMD=-1.16,95%CI:-1.90,-0.41** | **N=1, n1=17,n2=16, SMD=-0.99,95%CI:-1.72,-0.26** | N=1, n1=25,n2=21, RR=1.34,95%CI:0.52,3.49 | N=1, n1=25,n2=21, RR=5.90,95%CI:0.32,108.03 |
|  | Imipramine | NA | NA | N=1, n1=23,n2=18, SMD=-0.19,95%CI:-0.80,0.43 | N=1, n1=23,n2=18, SMD=-0.25,95%CI:-0.87,0.37 | N=1, n1=23,n2=18, SMD=-0.22,95%CI:-0.84,0.40 | N=1, n1=23,n2=18, SMD=-0.46,95%CI:-1.08,0.17 | N=1, n1=23,n2=18, RR=0.78,95%CI:0.47,1.30 | N=1, n1=23,n2=18, RR=1.04,95%CI:0.27,4.08 |
| TeCAs | Mirtazapine | **N=1, n1=17,n2=9, SMD=-1.87,95%CI:-2.85,-0.89** | NA | NA | NA | **N=1, n1=17,n2=9, SMD=-0.91,95%CI:-1.76,-0.05** | **N=1, n1=17,n2=9, SMD=-0.89,95%CI:-1.74,-0.04** | N=1, n1=17,n2=9, RR=0.53,95%CI:0.13,2.11 | N=1, n1=17,n2=9, RR=3.80,95%CI:0.22,66.09 |
| α2A receptor agonist | Guanfacine | N=1, n1=17,n2=9, SMD=-1.87,95%CI:-2.85,-0.89 | N=1, n1=18,n2=17, SMD=-0.23,95%CI:-0.89, 0.44 | N=1, n1=18,n2=17, SMD=-0.10,95%CI:-0.77, 0.56 | N=1, n1=18,n2=17, SMD=-0.38,95%CI:-1.05, 0.29 | N=2, n1=47,n2=51, SMD=-0.14,95%CI:-0.54, 0.26,I^2^=0% | NA | N=2, n1=47,n2=51, RR=2.15,95%CI:0.79,5.82,I^2^=0% | N=1, n1=29,n2=34, RR=8.19,95%CI:0.44,152.11 |

**Note:** MAOIs: Monoamine oxidase inhibitors, NDRIs: Norepinephrine and dopamine reuptake inhibitors, NK1: Neurokinin-1 receptor antagonist, NRI: Norepinephrine reuptake inhibitor, SARIs: Serotonin antagonist and reuptake inhibitors, SMSs: Serotonin modulator and stimulators, SNRIs: Serotonin and norepinephrine reuptake inhibitors, SSRIs: Selective serotonin reuptake inhibitors, TCAs: Tricyclic antidepressants, TeCAs: Tetracyclic antidepressants, NA: Not applicable.

**Supplemental Table 5** Active comparisons for efficacy and acceptability of pharmacological management for PTSD

| **Active drugs** | **PTSD total symptoms (clinician-rated)** | **Re-experiencing** | **Avoidance** | **Hyperarousal** | **Depression** | **Anxiety** | **All-cause discontinuation** | **Discontinuation due to adverse effect** |
| --- | --- | --- | --- | --- | --- | --- | --- | --- |
| Alpha blockers vs. Antihistamine | N=1,  n1=33,n2=34,  SMD=-0.25,95%CI:-0.73 to 0.24 | NA | NA | NA | NA | NA | NA | NA |
| NRIs vs. SSRIs | N=1,  n1=11,n2=17,  SMD=0.57,95%CI:-0.21 to 1.34 | N=1,  n1=11,n2=17,  SMD=0.40,95%CI:-0.37 to 1.16 | N=1,  n1=11,n2=17,  SMD=0.37,95%CI:-0.40 to 1.14 | N=1,  n1=11,n2=17,  SMD=0.47,95%CI:-0.30 to 1.24 | N=1,  n1=11,n2=17,  SMD=0.24,95%CI:-0.52 to 1.00 | N=1,  n1=11,n2=17,  SMD=0.00,95%CI:-0.76 to 0.76 | N=1,  n1=20,n2=20,  RR=3.00,95%CI:0.95 to 9.48 | N=1,  n1=20,n2=20,  RR=3.00,95%CI:0.95 to 9.48 |
| SARIs vs. SSRIs | N=1,  n1=13,n2=13,  SMD=-0.01,95%CI:-0.78 to 0.76 | N=1,  n1=13,n2=13,  SMD=-0.22,95%CI:-0.99 to 0.55 | N=1,  n1=13,n2=13,  SMD=0.46,95%CI:-0.32 to 1.24 | N=1,  n1=13,n2=13,  SMD=0.12,95%CI:-0.65 to 0.89 | N=1,  n1=13,n2=13,  SMD=-0.09,95%CI:-0.86 to 0.68 | N=1,  n1=13,n2=13,  SMD=-0.31,95%CI:-1.09 to 0.46 | NA | N=1,  n1=13,n2=13,  SRR=1.00,95%CI:0.16 to 6.07 |
| SNRIs vs. SSRIs | N=1,  n1=179,n2=173,  SMD=-0.07,95%CI:-0.28 to 0.14 | N=1,  n1=179,n2=173,  SMD=-0.07,95%CI:-0.28 to 0.14 | N=1,  n1=179,n2=173,  SMD=-0.02,95%CI:-0.23 to 0.19 | N=1,  n1=179,n2=173,  SMD=-0.07,95%CI:-0.28 to 0.14 | N=1,  n1=179,n2=173,  SMD=-0.09,95%CI:-0.30 to 0.11 | NA | N=1,  n1=179,n2=173,  RR=0.84,95%CI:0.62 to 1.14 | N=1,  n1=179,n2=173,  RR=0.75,95%CI:0.41 to 1.36 |
| MAOI vs. TCAs | NA | **N=1,**  **n1=19,n2=23,**  **SMD=-0.64,95%CI:-1.27 to -0.02** | N=1,  n1=19,n2=23,  SMD=-0.56,95%CI:-1.18 to 0.06 | NA | N=1,  n1=19,n2=23,  SMD=-0.14,95%CI:-0.75 to 0.47 | N=1,  n1=19,n2=23,  SMD=-0.30,95%CI:-0.91 to 0.32 | N=1,  n1=19,n2=23,  RR=0.40,95%CI:0.16 to 1.05 | N=1,  n1=19,n2=23,  RR=0.30,95%CI:0.04 to 2.48 |
| Citalopram vs. Sertraline | **N=1,**  **n1=25,n2=23,**  **SMD=0.65,95%CI:0.07 to 1.23** | N=1,  n1=25,n2=23,  SMD=0.14,95%CI:-0.43 to 0.71 | **N=1,**  **n1=25,n2=23,**  **SMD=0.93,95%CI:0.33 to 1.52** | N=1,  n1=25,n2=23,  SMD=0.51,95%CI:-0.06 to 1.09 | N=1,  n1=25,n2=23,  SMD=0.00,95%CI:-0.57 to 0.56 | NA | N=1,  n1=25,n2=23,  RR=0.77,95%CI:0.27 to 2.18 | NA |
| Nefazodone vs. Sertraline | N=1,  n1=13,n2=13,  SMD=-0.01,95%CI:-0.78 to 0.76 | N=1,  n1=13,n2=13,  SMD=-0.22,95%CI:-0.99 to 0.55 | N=1,  n1=13,n2=13,  SMD=0.46,95%CI:-0.32 to 1.24 | N=1,  n1=13,n2=13,  SMD=0.12,95%CI:-0.65 to 0.89 | N=1,  n1=13,n2=13,  SMD=-0.09,95%CI:-0.86 to 0.68 | N=1,  n1=13,n2=13,  SMD=-0.31,95%CI:-1.09 to 0.46 | NA | N=1,  n1=13,n2=13,  RR=1,95%CI:0.16 to 6.07 |
| Prazosin vs. Hydroxyzine | N=1,n1=33,n2=34,SMD=-0.25,95%CI:-0.73 to 0.24 | NA | NA | NA | NA | NA | NA | NA |
| Reboxetine vs. Fluvoxamine | N=1,  n1=11,n2=17,  SMD=0.57,95%CI:-0.21 to 1.34 | N=1,  n1=11,n2=17,  SMD=0.40,95%CI:-0.37 to 1.16 | N=1,  n1=11,n2=17,  SMD=0.37,95%CI:-0.40 to 1.14 | N=1,  n1=11,n2=17,  SMD=0.47,95%CI:-0.30 to 1.24 | N=1,  n1=11,n2=17,  SMD=0.24,95%CI:-0.52 to 1.00 | N=1,  n1=11,n2=17,  SMD=0.00,95%CI:-0.76 to 0.76 | N=1,  n1=20,n2=20,  RR=3.00,95%CI:0.95 to 9.48 | N=1,  n1=20,n2=20,  RR=3,95%CI:0.95 to 9.48 |
| Venlafaxine vs. Sertraline | N=1,  n1=179,n2=173,  SMD=-0.07,95%CI:-0.28 to 0.14 | N=1,  n1=179,n2=173,  SMD=-0.07,95%CI:-0.28 to 0.14 | N=1,  n1=179,n2=173,  SMD=-0.02,95%CI:-0.23 to 0.19 | N=1,  n1=179,n2=173,  SMD=-0.07,95%CI:-0.28 to 0.14 | N=1,  n1=179,n2=173,  SMD=-0.09,95%CI:-0.30 to 0.11 | NA | N=1,  n1=179,n2=173,  RR=0.84,95%CI:0.62 to 1.14 | N=1,  n1=179,n2=173,  RR=0.75,95%CI:0.41 to 1.36 |
| Phenelzine vs. Imipramine | NA | **N=1,**  **n1=19,n2=23,**  **SMD=-0.64,95%CI:-1.27 to -0.02** | N=1,  n1=19,n2=23,  SMD=-0.56,95%CI:-1.18 to 0.06 | NA | N=1,  n1=19,n2=23,  SMD=-0.14,95%CI:-0.75 to 0.47 | N=1,  n1=19,n2=23,  SMD=-0.30,95%CI:-0.91 to 0.32 | N=1,  n1=19,n2=23,  RR=-0.40,95%CI:0.16 to 1.05 | N=1,  n1=19,n2=23,  RR=0.30,95%CI:0.04 to 2.48 |

**Note:** MAOIs: Monoamine oxidase inhibitors, NRI: Norepinephrine reuptake inhibitor, SNRIs: Serotonin and norepinephrine reuptake inhibitors, SSRIs: Selective serotonin reuptake inhibitors, TCAs: Tricyclic antidepressants, NA: Not applicable.

**Supplemental Table 6** Publication bias using trim-and-fill method of placebo-controlled comparisons for efficacy and acceptability of pharmacological management in all outcomes

| **Outcomes** | **Active drugs vs. Placebo** | **Unadjusted pooled analysis results** | **Number of filled-in study** | **Adjusted pooled analysis results** |
| --- | --- | --- | --- | --- |
| PTSD total symptoms (clinician-rated) | Atypical antipsychotics | N=12, SMD=-0.30 [-0.46, -0.13] | 1 | SMD=-0.27 [-0.43, -0.11] |
|  | SSRIs | N=18, SMD=-0.33 [-0.40, -0.25] | 2 | SMD=-0.33 [-0.40, -0.26] |
| Reexperiencing | SSRIs | N=15, SMD=-0.37 [-0.49, -0.25] | 2 | SMD=-0.29 [-0.37, -0.21] |
| Avoidance | SSRIs | N=15, SMD=-0.28 [-0.38, -0.17] | 0 | SMD=-0.28 [-0.38, -0.17] |
| Hyperarousal | SSRIs | N=15, SMD=-0.34 [-0.41, -0.26] | 0 | SMD=-0.34 [-0.41, -0.26] |
| Depression | SSRIs | N=12, SMD=-0.28 [-0.41, -0.16] | 5 | SMD=-0.40 [-0.47, -0.33] |
| All-cause discontinuation rates | Anticonvulsants | N=10, RR=0.87 [0.67, 1.11] | 0 | RR=0.87 [0.67, 1.11] |
|  | Atypical antipsychotics | N=12, RR=1.07 [0.79, 1.46] | 4 | RR=0.83 [0.62, 1.11] |
|  | SSRIs | N=19, RR=0.99 [0.89, 1.10] | 0 | RR=0.99 [0.89, 1.10] |
| Discontinuation rate due to adverse effect | Anticonvulsants | N=10, RR=1.54 [0.91, 2.60] | 3 | RR=1.00 [0.61, 1.64] |
|  | Atypical antipsychotics | N=13, RR=2.06 [1.10, 3.84] | 3 | RR=1.57 [0.86, 2.87] |
|  | SSRIs | N=16, RR=1.37 [1.08, 1.74] | 0 | RR=1.37 [1.08, 1.74] |

**Note:** PTSD: Post-traumatic stress disorder, RR: Risk ratios, SSRIs: Selective serotonin reuptake inhibitors, SMD: Standardized mean difference. Since the number of studies included in the anxiety outcome is less than 10, there did perform the trim-and-fill method.
